# Diagnostic performance of deep learning in ultrasound diagnosis of breast cancer: a systematic review

**DOI:** 10.1038/s41698-024-00514-z

**Published:** 2024-01-27

**Authors:** Qing Dan, Ziting Xu, Hannah Burrows, Jennifer Bissram, Jeffrey S. A. Stringer, Yingjia Li

**Affiliations:** 1 Department of Ultrasound, Nanfang Hospital, Southern Medical University, 510515 Guangzhou, China; 2 Global Women’s Health, The University of North Carolina at Chapel Hill, Chapel Hill, NC 27599 USA; 3 Health Sciences Library, The University of North Carolina at Chapel Hill, Chapel Hill, NC 27599 USA

**Keywords:** Diagnosis, Breast cancer

## Abstract

Deep learning (DL) has been widely investigated in breast ultrasound (US) for distinguishing between benign and malignant breast masses. This systematic review of test diagnosis aims to examine the accuracy of DL, compared to human readers, for the diagnosis of breast cancer in the US under clinical settings. Our literature search included records from databases including PubMed, Embase, Scopus, and Cochrane Library. Test accuracy outcomes were synthesized to compare the diagnostic performance of DL and human readers as well as to evaluate the assistive role of DL to human readers. A total of 16 studies involving 9238 female participants were included. There were no prospective studies comparing the test accuracy of DL versus human readers in clinical workflows. Diagnostic test results varied across the included studies. In 14 studies employing standalone DL systems, DL showed significantly lower sensitivities in 5 studies with comparable specificities and outperformed human readers at higher specificities in another 4 studies; in the remaining studies, DL models and human readers showed equivalent test outcomes. In 12 studies that assessed assistive DL systems, no studies proved the assistive role of DL in the overall diagnostic performance of human readers. Current evidence is insufficient to conclude that DL outperforms human readers or enhances the accuracy of diagnostic breast US in a clinical setting. Standardization of study methodologies is required to improve the reproducibility and generalizability of DL research, which will aid in clinical translation and application.

## Introduction

Breast cancer is the world’s most prevalent cancer and remains the major cause of cancer-associated deaths globally. GLOBCAN estimated that in 2020, there were about 2.3 million women diagnosed with breast cancer and 685,000 breast cancer-associated deaths worldwide^1^. Early and accurate diagnosis results in better patient outcomes. Breast ultrasound (US) is low-cost, easy-to-operate, radiation-free, portable, and typically helpful for distinguishing between a cystic and a solid breast mass. The effectiveness of the US as a diagnostic tool for palpable breast abnormalities is widely recognized, especially in cases involving dense breast tissues or mammographically occult lesions^2–4^. Additionally, the US is considered the preferred imaging method for providing guidance during breast biopsy procedures^5,6^. However, the diagnostic efficacy and reproducibility of US examinations are relatively low due to their dependence on the knowledge and experience of the operators^7,8^.

**Fig. 1:**
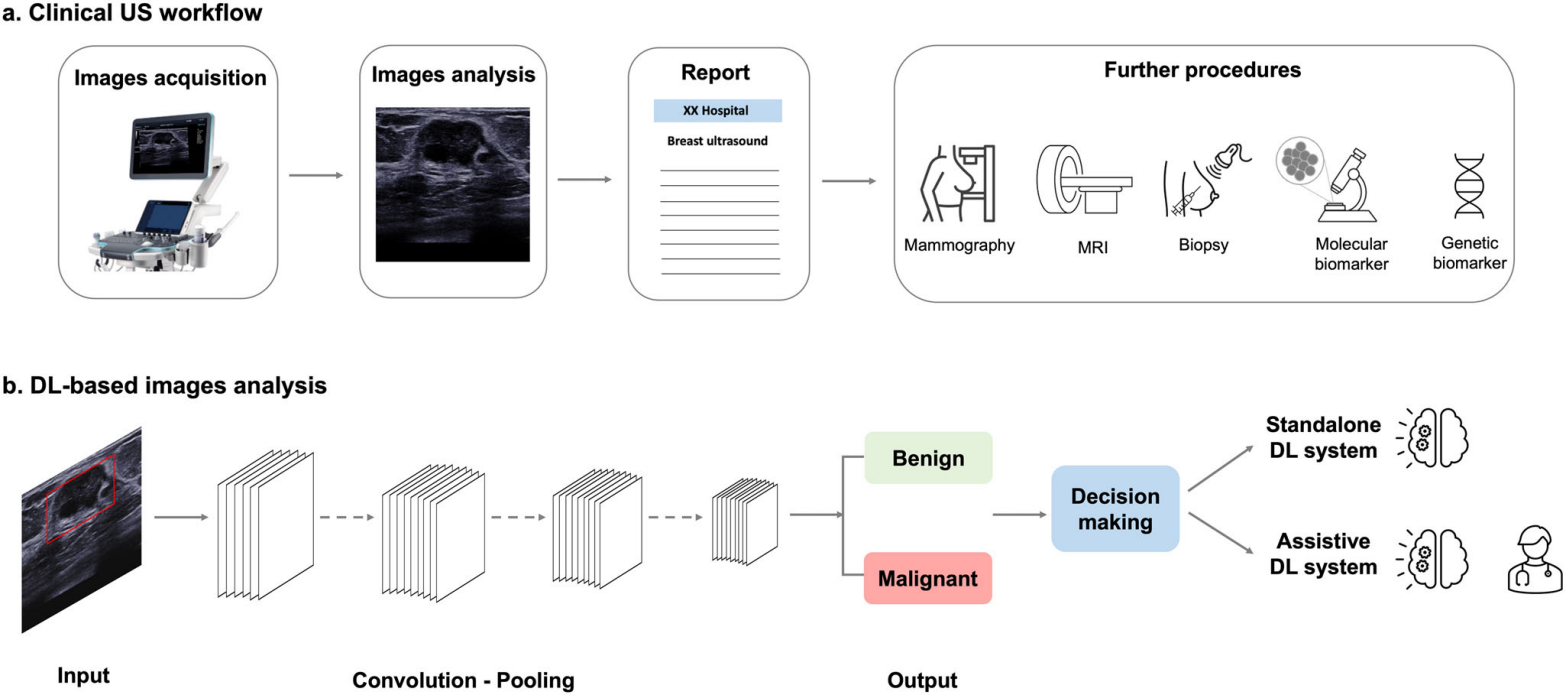
Schematic illustration of clinical US examination workflow and the image-related task where DL-based system could have a large impact. **a** Clinical US workflow comprises image acquisition, image analysis (which may involve DL), report generation, and further procedures based on diagnostic reports. **b** A DL system comprises multiple layers where feature extraction, selection, and ultimate classification are performed simultaneously during training. US images as input are analyzed and the DL model gives binary classification (benign or malignant). Final assessment is made based on the decision of the DL system alone or in combination with human radiologists.

Deep learning (DL), an innovative artificial intelligence (AI) technology, excels at image-related tasks, including abnormities detection, segmentation, and classification (Fig. 1). The integration of DL into the US imaging workflow offers numerous benefits, including improved efficiency, reduced errors, and automated quantitative assessments^9^. Consequently, significant efforts have been made to facilitate the clinical application of DL in medical imaging. For instance, the DL-based ultrasonography system known as S-Detect (Samsung Medison, Seoul, Korea) has gained increasing popularity for breast cancer diagnosis. This system enables automatic segmentation and interpretation of US morphological descriptions, providing a dichotomous classification (possibly benign or possibly malignant) that serves as a reference for radiologists during the final diagnostic process^10^.

Several recent reports have suggested that DL-based interpretation of breast US is on par with or even superior to that of a human radiologist^11–15^. However, the application of DL in clinical practice remains controversial and results vary across different studies. Current reviews^10,16^ focused on evaluating the application potentials of commercial products, such as S-Detect. There is a paucity of evidence-based systematic reviews specific to the general diagnostic performance of employing DL models in clinical practice of breast US, in particular comprehensive comparison between DL and human readers. Our work aims to assess current evidence on the diagnostic performance of DL algorithms in the detection and classification of breast lesions in clinical US tests, including (1) whether standalone DL systems outperform radiologists in breast cancer diagnosis and (2) whether assistive DL systems can improve diagnostic performance when used in concert with human radiologists.

## Results

### Study selection and study characteristics

**Fig. 2:**
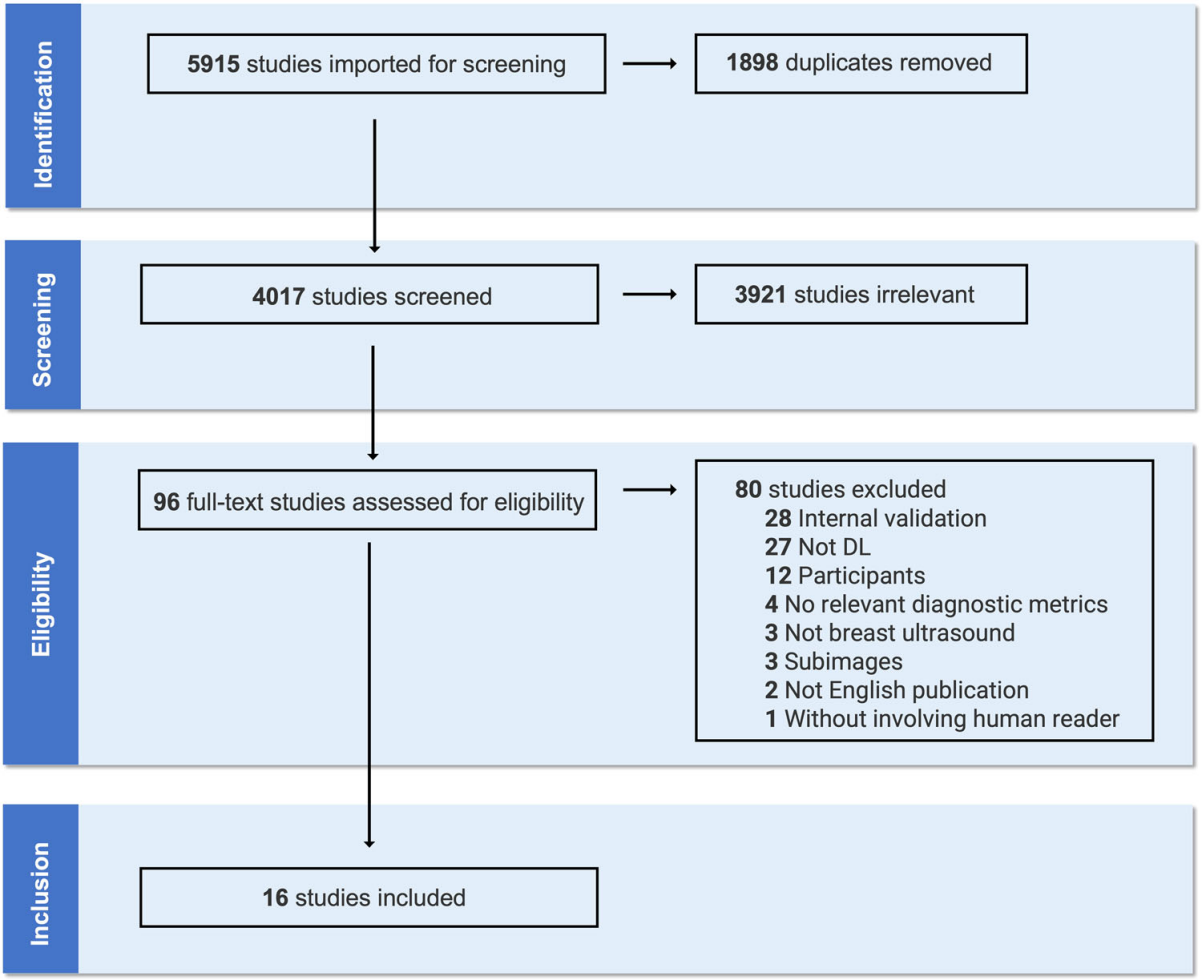
PRISMA diagram of included and excluded studies at each stage of the review. Sixteen publications were included in the database (PubMed, Embase, Scopus, and Cochrane Library) after removing duplicates, irrelevant studies, and studies that did not meet the inclusion criteria.

Database searches initially yielded 4017 unique results after removing 1898 duplicates, among which 96 potentially eligible studies were further reviewed through full texts. Overall, as shown in Fig. 2, 16 studies^17–32^ were ultimately included in this review, according to inclusion criteria. In addition, based on the PICO framework (population, intervention, comparison, outcome), exclusions and the corresponding reasons after full-text review were presented in Supplementary Tables 1 and 2.

The main characteristics of the included 16 publications, including 14 studies using standalone DL systems and 12 studies using assistive DL systems, were presented in Table 1, Supplementary Tables 3 and 4, and Supplementary Fig. 1. These studies comprised 9238 women in total, of which 3 studies^30–32^ recruited 901, 582, and 5012 female participants respectively, the remaining 13 studies^17–29^ included smaller numbers of women (from 40 to 472). Seven studies evaluated data from China^19,21,23,27,29,30,32^, 6 studies enrolled participants from Korea^17,18,20,24,25,28^, 2 from Italy^22,26^, and the remaining 1 study^31^ used public multisite data from which the countries were not reported. Of all studies, 15 were conducted in a diagnostic setting, while the remaining 1 was evaluated in a screening setting^18^. All included studies employed DL convolutional neural networks, of which 14 were commercial DL systems, including S-Detect^17–26,28–30^ and BU-CAD^27^, and 2 were investigator-derived DL systems^31,32^. In addition, there were 6 studies^17,18,24–28^ using retrospective US images to compare the diagnostic accuracy of DL systems and human readers. For prospective test accuracy studies, multiple reader multiple case studies were performed under laboratory conditions^19–23,29,30,32^, without any randomized controlled trials or cohort studies based on real-world settings. Nine publications^17–20,22,27,29–31^ followed the fifth edition of Breast Imaging Reporting and Data System (BI-RADS) to make the final assessment, another 7 articles did not specify which version was used. BIRAD-4a was clearly described as the cutoff value in 13 studies^17–22,24,25,27–29,31,32^, while 2 studies^23,26^ using BIRADS-4b as the cutoff value. Another study^30^ evaluated the diagnostic accuracy using BIRAD-4a and BIRADS-4b as cut-off values, respectively. All studies used pathology as the gold standard, among which 7 studies^20,22,24,25,27,28,31^ employed follow-up as a supplement to the reference standard.

**Table 1:** Characteristics of 14 studies using standalone DL systems and 12 studies using assistive DL systems.

Study	Design	Site	Patient	US vendor^‡^	Index test	Comparator	BIRADS lexicon	Cutoff value	Reference standard
*Standalone DL system*									
Kim 2021^18^	Retrospective	1 (Korea)	146	RS85 Prestige	S-Detect	2 human readers (1 with 6-year experience, 1 with 3-month experience in breast imaging)	5th	4a	Pathology
Xiao 2019^19^	Prospective	1 (China)	437	RS80 Prestige	S-Detect	2 human readers (1 experienced, 1 resident)	5th	4a	Pathology
Cho 2018^20^	Prospective	1 (Korea)	116	RS80A	S-Detect	2 human readers (1 with 7-year experience, 1 with 1-year experience in breast imaging)	5th	4a	Pathology Follow up (>2 year)
Segni 2018^22^	Prospective	1 (Italy)	61	RS80A	S-Detect	5 human readers (1 experienced, 4 in-training residents)	5th	4a	Pathology Follow up (<2 years)
Xia 2021^23^	Prospective	1 (China)	40	RS80A	S-Detect	4 human readers (2 senior, 2 junior)	NR†	4b	Pathology
Lee 2022^24^	Retrospective	1 (Korea)	472	RS80A	S-Detect	6 human readers (3 experienced, 3 inexperience)	NR	4a	Pathology Follow up (>2 year)
Choi 2019^25^	Retrospective	1 (Korea)	226	RS80A	S-Detect	4 human readers (2 senior, 2 junior)	NR	4a	Pathology Follow up (7–28 months)
Nicosia 2022^26^	Retrospective	1 (Italy)	210	RS80A	S-Detect	4 human readers (2 senior, 2 junior)	NR	4b	Pathology
Lai 2022^27^	Retrospective	1 (China)	172	Philips iU22 Toshiba Aplio 500 Canon Aplio i800	BU-CAD	16 human readers	5th	4a	Pathology Follow up (>2 year)
Lee 2019^28^	Retrospective	1 (Korea)	413	RS80A	S-Detect	10 human readers (5 experienced, 5 inexperience)	NR	4a	Pathology Follow up (>1 year)
Wei 2021^29^	Prospective	1 (China)	192	RS80A	S-Detect	4 human readers (2 experienced, 2 inexperience)	5th	4a	Pathology
Wei 2022^30^	Prospective	9 (China)	901	RS80A	S-Detect	4 human readers (2 experienced, 2 inexperience)	5th	4a 4b	Pathology
Ciritsis 2019^31^	Retrospective	Training set: 1 site (country NR†) Test set: public dataset (multiple sites)	582	NR	In-house	2 human readers (over 8 years of experience)	5th	4a	Pathology Follow up (time NR†)
Gu 2022^32^	Prospective	Training set: 32 sites (China) Test set: public dataset (multiple sites)	5012	Resona7 Resona7s Resona7T Resona8 Resona8T DC-80	In-house	5 human readers (2 experienced, 3 inexperience)	NR	4a	Pathology
*Assistive DL system*									

Park 2019^17^	Retrospective	1 China	91	RS80A	Human readers + S-Detect	5 human readers (3 less experienced, 2 experienced)	5th	4a	PathologyFollow up (6–35 months)
Kim 2021^18^	Retrospective	1 (Korea)	146	RS85 Prestige	Human readers + S-Detect	2 human readers (with 6-year and 3-month experience in breast imaging, respectively)	5th	4a	Pathology
Cho 2018^20^	Prospective	1 (Korea)	116	RS80A	Human readers + S-Detect	2 human readers (with 7- and 1 1-year experience in breast imaging, respectively)	5th	4a	Pathology Follow up (>2 year)
Wang 2021^21^	Prospective	1 (China)	167	RS80A	Human readers + S-Detect	2 human readers (with 8- and 10-year experience in breast US, respectively)	NR	4a	Pathology
Xia 2021^23^	Prospective	1 (China)	40	RS80A	Human readers + S-Detect	4 human readers (2 senior, 2 junior)	NR	4b	Pathology
Lee 2022^24^	Retrospective	1 (Korea)	472	RS80A	Human readers + S-Detect	6 human readers (3 experienced, 3 inexperience)	NR	4a	Pathology Follow up (>2 year)
Choi 2019^25^	Retrospective	1 (Korea)	226	RS80A	Human readers + S-Detect	4 human readers (2 senior, 2 junior)	NR	4a	Pathology Follow up (7–28 months)
Lai 2022^27^	Retrospective	1 (China)	172	Philips iU22 Toshiba Aplio 500 Canon Aplio i800	Human readers + BU-CAD	16 human readers	5th	4a	Pathology Follow up (>2 year)
Lee 2019^28^	Retrospective	1 (Korea)	413	RS80A	Human readers + S-Detect	10 human readers (5 experienced, 5 inexperience)	NR	4a	Pathology Follow up (>1 year)
Wei 2021^29^	Prospective	1 (China)	192	RS80A	Human readers + S-Detect	4 human readers (2 experienced, 2 inexperience)	5th	4a	Pathology
Wei 2022^30^	Prospective	9 (China)	901	RS80A	Human readers + S-Detect	4 human readers (2 experienced, 2 inexperience)	5th	4a 4b	Pathology
Gu 2022^32^	Prospective	Training set: 32 sites (China) Test set: public dataset (multiple sites)	5012	Resona7 Resona7s Resona7T Resona8 Resona8T DC-80	Human readers + In-house AI system	5 human readers (2 experienced, 3 inexperience)	NR	4a	Pathology

US vendor^‡^: detailed information presented in Table S6. NR†: not reported.

### Diagnostic performance comparison

DL can function either as a standalone system where the algorithms independently generate diagnostic decisions, or as an assistant to radiologists where the final diagnosis is made by radiologists considering the DL outcomes. Consequently, the development of a successful DL product necessitates not only the construction of robust DL algorithms but also the exploration of how the algorithm outputs can enhance radiologists’ diagnostic capabilities. It is crucial to investigate the usefulness of DL outputs for radiologists, quantify the benefits of DL in patient care, and determine strategies to optimize these advantages.

In test accuracy comparison between DL systems and human readers, 4 studies evaluated the diagnostic performance of DL systems as standalone^19,22,26,31^, 2 studies employed assistive DL systems^17,21^, and another 10 studies assessed the roles of DL systems as both standalone and assistive systems^18,20,23–25,27–30,32^. Those studies employed human readers at various levels of clinical experiences in breast US and investigated the performance of DL systems compared to experienced and less experienced human readers.

### Standalone DL systems

In 14 studies using DL as a standalone system, the diagnostic accuracy of DL and human readers was compared (Table 2). In a study^20^ conducted by Cho et al. found DL had lower AUC than human readers. Two studies^22,24^ showed DL was equivalent to human readers in AUC. In contrast, another study^32^ reported a higher AUC of DL than human readers. More specifically, DL had superior AUC over less experienced human readers while comparable to experienced human readers in three studies^19,24,29^. As for accuracy, DL systems were more accurate than all human readers in two studies^24,32^. Wei et al.^29^ reported that DL was more accurate than less experienced human readers while comparable to experienced human readers. In contrast, another study showed DL was equivalent to less experienced human readers while more accurate than experienced human readers. In addition, standalone DL had lower sensitivity than overall human readers in five studies^19,20,24,30,32^. Another two studies^26,28^ found that DL was more sensitive than less experienced human readers but less sensitive than experienced human readers. In four studies^19,20,24,32^, DL exhibited higher specificity than overall human readers. In another study^26^, DL was more specific than less experienced human readers but less specific than experienced human readers. The remaining studies did not report comparable diagnostic measures between DL systems and human readers.

**Table 2:** Test outcomes of standalone and assistive DL systems.

Study	Index test/comparator	AUC (95% CI)	*P*_ΔAUC_	%Acc (95% CI)	*P*_ΔAcc_	%Sen (95% CI)	*P*_ΔSen_	%Spe (95% CI)	*P*_ΔSpec_
*Standalone DL system*									
Kim 2021^18^	DL	0.575		NR		30		84.9	
	Reader 1	0.545	NR	NR	NR	100	NR	8.9	NR
	Reader 2	0.541	NR	NR	NR	100	NR	8.2	NR
	Reader 3	0.545	NR	NR	NR	100	NR	8.9	NR
Xiao 2019^19^	DL	0.81 (0.77–0.85)		NR		85.32 (79.91–89.74)		76.96 (70.97–82.24)	
	Less experienced reader	0.7 (0.65–0.74)	<0.0001	NR	NR	92.2 (87.81–95.39)	<0.05	46.96 (40.37–53.63)	<0.05
	Experienced reader	0.81(0.77–0.84)	NS	NR	NR	98.62 (96.03–99.72)	<0.05	63.04 (56.45–69.29)	<0.05
Cho 2018^20^	DL	0.815 (0.745–0.885)		82.4 (75.5–89.2)		72.2 (60.3–84.2)		90.8 (83.7–97.8)	
	Less experienced reader	0.901 (0.846–0.956)	0.004	73.1 (65.1–81.1)	0.06	94.4 (88.3–100.0)	<0.001	55.4 (43.3–67.5)	<0.001
	Experienced reader	0.887 (0.826–0.947)	0.023	69.8 (61.5–78.0)	0.014	94.4 (88.3–100.0)	<0.001	49.2 (37.1–61.4)	<0.001
Segni 2018^22^	DL	0.82 (0.71–0.91)		NR		91.1 (78.8– 97.5)		70.8 (48.9– 87.4)	
	Less experienced reader								
	1	0.76 (0.66–0.86)	0	NR	NR	97.7 (88–99.9)	NR	54.2 (32.8–74.4)	NR
	2	0.83 (0.73–0.93)	0.831	NR	NR	95.5 (84.5–99.4)	NR	70.8 (48.9–87.4)	NR
	3	0.74 (0.63–0.84)	0.151	NR	NR	97.8 (88.2–99.9)	NR	50 (29.1–70.9)	NR
	4	0.75 (0.65–0.85)	0.206	NR	NR	100 (92–100)	NR	50 (29.1–70.9)	NR
	Experienced reader	0.84 (0.74 –0.94)	0.751	NR	NR	93.2 (81.3– 98.6)	NR	75.0 (53.3–90.2)	NR
Xia 2021^23^	DL	0.948		89.6		95.8		93.8	
	Reader	0.719	NR	43.8	NR	75	NR	68.8	NR
Lee 2022^24^	DL	0.855 (0.825‒0.886)		85.4 (82.2‒88.1)		86.1 (80.7‒90.1)		84.9 (80.6‒88.4)	
Lee 2022^24^	Reader	0.895 (0.854‒0.936)	0.05	72.4 (69.1‒75.4)	<0.001	95.4 (93.0‒97.0)	<0.001	56.6 (52.2‒60.8)	<0.001
Choi 2019^25^	DL	NR		92.1		85		95.4	
	Less experienced reader								
	1	NR	NR	79.4	NR	88.8	NR	75.1	NR
	2	NR	NR	88.9	NR	81.3	NR	92.5	NR
	Experienced reader								
	1	NR	NR	77.9	NR	88.8	NR	72.8	NR
	2	NR	NR	84.2	NR	86.3	NR	83.2	NR
Nicosia 2022^26^	DL	NR		NR		85.2		79.8	
	Less experienced reader								
	1	NR	NR	NR	NR	75.4	<0.001	68.4	0.001
	2	NR	NR	NR	NR	75.4	<0.001	65.8	<0.001
	Experienced reader								
	1	NR	NR	NR	NR	94.4	<0.001	86.8	0.08
	2	NR	NR	NR	NR	95.8	<0.001	85.1	0.34
Lai 2022^27^	DL	0.8591		94.77		96.92		55.14	
	Average readers	0.7582 (0.7014–0.8151)	NR	NR	NR	95.77 (90.88– 100.66)	NR	24.07 (15.97–32.17)	NR
Lee 2019^28^	DL	0.73 (0.67–0.78)		NR		76 (65–86)		69 (65–74)	
	Less experienced reader	0.65 (0.58–0.71)	0.013	NR	NR	59 (46–71)	0.007	70 (66–75)	0.71
	DL	0.79 (0.74–0.84)		NR		81 (70–89)		77 (73–81)	
	Experienced reader	0.83 (0.8–0.86)	0.101	NR	NR	97 (90–100)	<0.001	70 (65–74)	0.004
Wei 2021^29^	DL	0.874		88.3		85.5		89.3	
	Less experienced reader								
	1	0.735	<0.001	73.3	<0.001	73.9	0.057	73.1	<0.001
	2	0.802	0.014	80.5	0.005	79.7	0.388	80.7	0.009
	Experienced reader								
	1	0.843	0.255	87.2	0.749	78.3	0.227	90.4	0.851
	2	0.901	0.113	91	0.146	88.4	0.625	91.9	0.267
Wei 2022^30^	DL	0.906 (0.885–0.924)		89.6 (87.4– 91.4)		94.2 (91.0– 96.5)		87.0 (83.9– 89.6)	
Wei 2022^30^	Less experienced readers^a^	0.696 (0.665–0.726)	<0.001	61.6 (58.4–64.7)	<0.001	98.5 (96.5–99.5)	<0.001	40.7 (36.7–44.8)	<0.001
	Less experienced readers^b^	0.874 (0.850–0.895)	0.007	85.9 (83.5–88.0)	0.005	89.6 (85.7–92.7)	0.458	82.1 (78.7–85.1)	0.007
	Experienced readers^a^	0.734 (0.704–0.763)	<0.001	66.5 (63.3–69.5)	<0.001	98.5 (96.5–99.5)	0.001	48.4 (44.2–52.5)	<0.001
	Experienced readers^b^	0.883 (0.860–0.903)	0.057	87.9 (85.6–89.9)	0.21	92.6 (89.2–95.2)	0.014	87.0 (83.9– 89.6)	>0.999
Ciritsis 2019^31^	DL	0.967 (0.86–0.99)		NR		89.47		100	
	Reader 1	0.938 (0.82– 0.99)	NR	NR	NR	100	NR	87.5	NR
	Reader 2	0.88 (0.74– 0.96)	NR	NR	NR	84.21	NR	95.83	NR
Gu 2022^32^	DL	0.924 (0.879–0.957)		85.57 (79.94–90.12)		89.77 (81.47–95.22)		82.30 (74.00–88.84)	
	Readers	0.843 (0.819–0.865)	<0.0001	66.27(63.25– 69.19)	<0.0001	96.82 (94.72–98.25)	<0.0001	42.48 (38.36–46.67)	<0.0001
*Assistive DL system*									
Park 2019^17^	Less experienced								
	Reader 1 + DL	0.828 (0.745–0.912)		54		97.6		23.7	
	Reader 1	0.623 (0.501–0.746)	<0.001	43	0.03	65.9	<0.001	27.1	0.56
	Reader 2 + DL	0.823 (0.742–0.904)		74		85.4		66.1	
	Reader 2	0.702 (0.596–0.808)	0.001	61	0.008	75.6	0.1	50.8	0.04
	Reader 3 + DL	0.839 (0.762–0.917)		58		97.6		30.5	
	Reader 3	0.759 (0.660–0.859)	0.04	51	0.15	87.8	0.05	27.1	0.59
	Experienced								
	Reader 1 + DL	0.907 (0.848–0.967)		74		90.2		66.1	
Park 2019^17^	Reader 1	0.856 (0.776–0.936)	0.02	66	0.006	85.4	0.16	52.5	0.02
	Reader 2 + DL	0.904 (0.837–0.971)		76		90.2		66.1	
	Reader 2	0.889 (0.821–0.957)	0.16	70	0.05	92.7	0.327	54.2	0.02
Kim 2021^18^	Reader 1 + DL	0.803		NR		90		70.5	
	Reader 1	0.545	<0.001	NR	NR	100	>0.999	8.9	<0.001
	Reader 2 + DL	0.658		NR		100		31.5	
	Reader 2	0.541	<0.001	NR	NR	100	NA	8.2	<0.001
	Reader 3 + DL	0.758		NR		90		61.6	
	Reader 3	0.545	<0.001	NR	NR	100	>0.999	8.9	<0.001
Cho 2018^20^	Less experienced reader + DL	0.895 (0.835–0.956)		86.6 (80.4–92.7)		87.0 (78.1–96.0)		86.2 (77.8–94.6)	
	Less experienced reader	0.887 (0.826–0.947)	>0.999	69.8 (61.5–78.0)	<0.001	94.4 (88.3–100.0)	0.17	49.2 (37.1–61.4)	<0.001
	Experienced reader + DL	0.901(0.844–0.958)		85.7 (79.4–92.0)		94.4 (88.3–100.0)		87.7 (79.7–95.7)	
	Experienced reader	0.901 (0.846–0.956)	>0.999	73.1 (65.1–81.1)	0.015	83.3 (73.4–93.3)	0.04	55.4 (43.3–67.5)	<0.001
Wang 2021^21^	Readers + DL^c^	0.777 (0.707–0.847)	0.08	75.7 (68.8–81.5)	0.095	97.4 (90.2–99.6)	1	57.9 (47.3–67.8)	0.042
	Readers + DL^d^	0.822 (0.757–0.886)	0.01	80.9 (74.4–86.1)	0.005	94.9 (86.7–98.3)	0.681	69.4 (59.1–78.3)	<0.001
	Readers	0.703 (0.626–0.780)		67.6 (60.3–74.2)		97.4 (90.2–99.6)		43.2 (33.2–53.8)	
Xia 2021^23^	Less experienced reader + DL	0.948		89.6		95.8		93.8	
	Less experienced reader	0.719	NR	43.8	NR	75	NR	68.8	NR
	Experienced reader + DL	0.969		93.8		100		93.8	
	Experienced reader	0.802	NR	60.5	NR	79.2	NR	81.3	NR
Lee 2022^24^	Readers + DL^e^	0.908 (0.876‒0.941)	0.093	75.3 (72.2‒78.2)	<0.001	95.2 (92.4‒97.0)	0.725	61.8 (57.5‒65.8)	<0.001
Lee 2022^24^	Readers + DL^f^	0.913 (0.886‒0.941)	0.099	79.0 (76.0‒81.6)	0.001	93.8 (90.7‒96.0)	0.087	68.8 (64.7‒72.6)	0.001
	Readers	0.895 (0.854‒0.936)		72.4 (69.1‒75.4)		95.4 (93.0‒97.0)		56.6 (52.2‒60.8)	
Choi 2019^25^	Less experienced reader 1 + DL	0.951		86.2		95		82.1	
	Less experienced reader 1	0.906	NR	79.4	0.045	88.8	0.182	75.1	0.014
	Less experienced reader 2 + DL	0.914		88.1		86.3		89	
	Less experienced reader 2	0.895	NR	88.9	0.78	81.3	0.221	92.5	0.211
	Experienced reader 1 + DL	0.919		90.9		86.3		93.1	
	Experienced reader 1	0.884	NR	77.9	<0.001	88.8	0.683	72.8	<0.001
	Experienced reader 2 + DL	0.942		90.1		90		90.2	
	Experienced reader 2	0.919	NR	84.2	0.046	86.3	0.371	83.2	0.006
Lai 2022^27^	Readers + DL	0.8294 (0.7777– 0.8813)		NR		98.17 (0.9492– 1.0143)		30.67 (21.93–39.40)	
	Readers	0.7582 (0.7014–0.8151)	<0.0001	NR	NR	95.77 (90.88–10.066)	0.2991	24.07 (15.97–32.17)	0.0448
Lee 2019^28^	Less experienced readers + DL	0.71 (0.65–0.77)		NR		69 (57–80)		73 (69–77)	
	Less experienced readers	0.65 (0.58–0.71)	0.001	NR	NR	59 (46–71)	0.008	70 (66–75)	0.033
	Experienced readers + DL	0.84 (0.81–0.87)		NR		96 (88–99)		72 (68–77)	
	Experienced readers	0.83 (0.8–0.86)	0.451	NR	NR	97 (90–100)	0.317	70 (65–74)	0.003
Wei 2021^29^	Reader 1 + DL	0.875		89.1		84.1		90.9	
	Reader 1	0.735	<0.001	73.3	<0.001	73.9	0.039	73.1	<0.001
	Reader 2 + DL	0.867		87.2		85.5		87.8	
Wei 2021^29^	Reader 2	0.802	<0.001	80.5	<0.001	79.7	0.125	80.7	0.001
	Reader 3 + DL	0.872		89.5		82.6		91.9	
	Reader 3	0.843	0.099	87.2	0.181	78.3	0.375	90.4	0.508
	Reader 4 + DL	0.901		91		88.4		91.9	
	Reader 4	0.901	>0.999	91	>0.999	88.4	>0.999	91.9	>0.999
Wei 2022^30^	Less experienced readers + DL	0.87 (0.85–0.89)		NR		97.24 (96.17–98.31)		40.7 (37.49–43.9)	
	Less experienced readers	0.7 (0.66–0.73)	NR	NR	NR	98.47 (97.66–99.27)	NR	77.22 (74.48–79.96)	NR
	Experienced readers + DL	0.89 (0.87–0.91)		NR		96.32 (95.09–97.55)		81.39 (78.85–83.93)	
	Experienced readers	0.73 (0.70–0.76)	NR	NR	NR	98.47 (97.66–99.27)	NR	48.35 (45.08–51.61)	NR
Gu 2022^32^	Readers + DL^g^	0.861 (0.838–0.881)	<0.0001	78.71 (76.04– 81.20)	<0.0001	97.27 (95.28–98.58)	0.8036	64.25 (60.14–68.21)	<0.0001
	Readers + DL^h^	0.908 (0.888–0.925)	<0.0001	80.40 (77.81– 82.81)	<0.0001	97.73 (95.86–98.91)	0.4545	66.90 (62.85–70.77)	<0.0001
	Readers	0.843 (0.819–0.865)		66.27 (63.25– 69.19)		96.82 (94.72–98.25)		42.48 (38.36–46.67)	

*Acc* accuracy, *Sen* sensitivity, *Spe* specificity, *NS* not significant, *NA* not applicable.

^a^Category 4a as the cut-off value.

^b^Category 4b as the cut-off value^12^.

^c^If both the assessments of longitudinal and transverse sections from the DL model were possibly benign, the final BIRADS category would be downgraded.

^d^If any of the assessments from DL were possibly benign, the final BIRADS category would be downgraded^16^.

^e^Sequential reading mode.

^f^Simultaneous reading mode^6^.

^g^If the DL model assessed the lesion as malignant or benign, the final BIRADS classification would be upgraded or downgraded by one level.

^h^The BIRADS assessment was flexibly adjusted by human readers after combining DL’s outcomes.

### Assistive DL systems

In 12 studies that assessed assistive DL systems (Table 2), three studies^18,27,32^ reported improved AUC of human readers when combining with DL systems. Another study^20^ showed assistive DL had a comparable AUC to human readers alone. To investigate the assistive effects of DL on human readers with different experiences, two studies^17,24^ found that assistive DL systems had higher AUC than less experienced human readers but the positive impacts did not work for experienced human readers. In accuracy tests, assistive DL systems were more accurate than human readers in three studies^20,24,32^. However, no studies showed improved overall sensitivity of the combination of DL and human readers compared to human readers alone. One study^28^ reported improved sensitivity of an assistive DL system compared to less experienced human readers but this advantage was not maintained when used by experienced human readers. Improved specificity in overall human readers was reported in seven studies^18,20,21,24,27,28,32^ that used assistive DL systems. Interestingly, in a study^17^ reported by Park and coworkers, the assistive DL technology improved diagnostic specificity among experienced human readers but not among inexperienced readers. While in another study^20^, less experienced human readers were aided in terms of specificity by the assistive DL system.

**Fig. 3:**
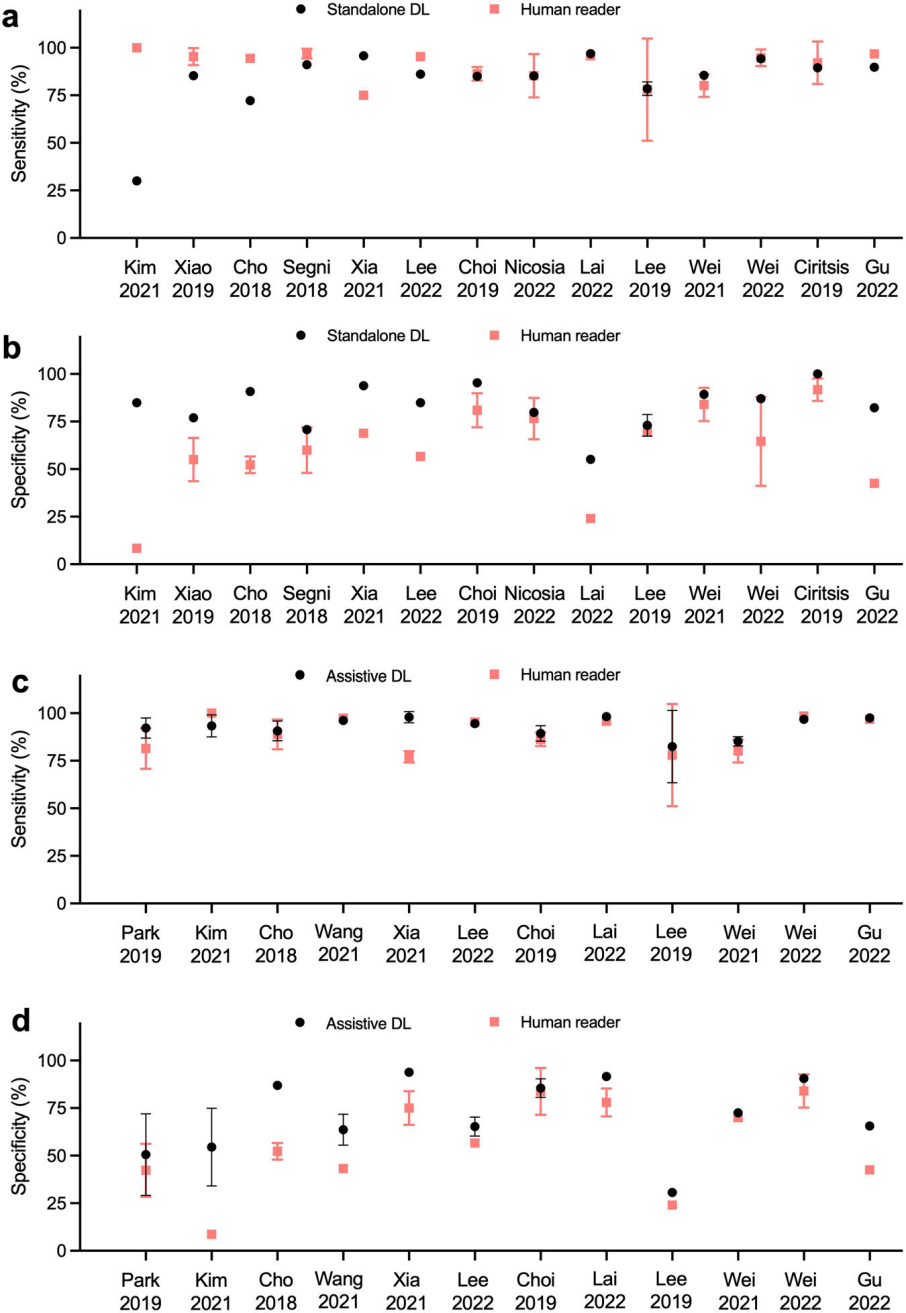
Estimated sensitivity and specificity of standalone/assistive DL systems and human readers. **a** Sensitivities of standalone DL systems and average human readers. **b** Specificities of standalone DL systems and average human readers. **c** Sensitivities of assistive DL systems and average human readers. **d** Specificities of assistive DL systems and average human readers. Error bar represents SD.

In Fig. 3, we estimated the sensitivity and specificity of DL systems and average human readers. We tentatively infer both standalone and assistive DL systems are more specific than average human readers while whether they are more sensitive remains unclear. However, complete 2 × 2 contingency tables were not available in most studies so that we were unable to conduct a thorough diagnostic analysis for all included studies.

### Quality assessment

**Fig. 4:**
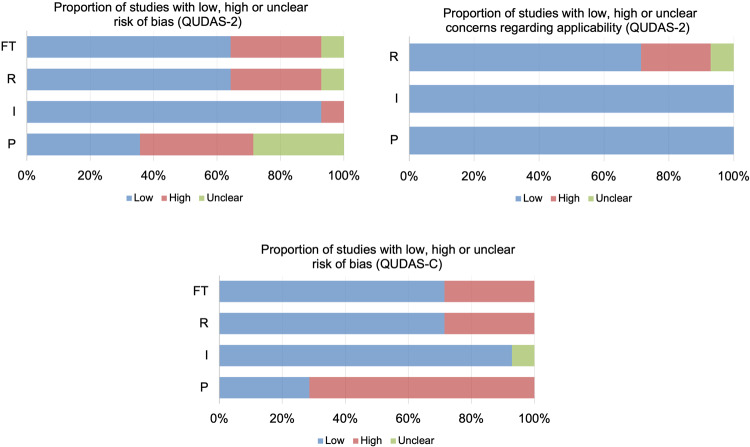
Graphic display of QUDAS-2 and QUDAS-C for studies using standalone DL systems. The proportion of studies with low, high, unclear risk of bias and concerns regarding applicability.

**Fig. 5:**
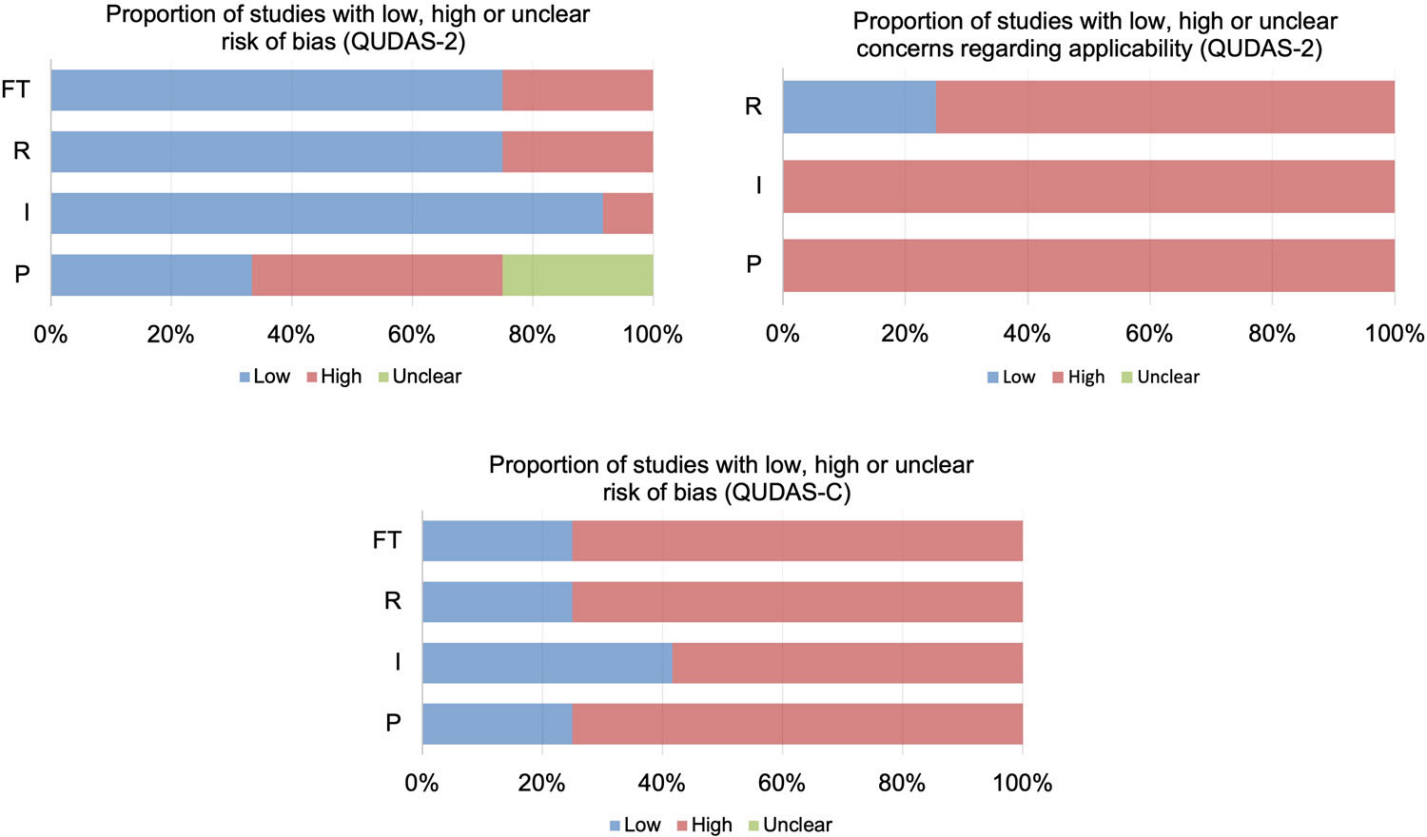
Graphic display of QUDAS-2 and QUDAS-C for studies using assistive DL systems. The proportion of studies with low, high, unclear risk of bias and concerns regarding applicability.

Based on QUADAS-2 and QUADAS-C tools, we tailored the signal questions in four domains, including patient selection, index tests, reference standard, flow, and timing, to assess the quality and applicability of included studies (Supplementary Table 5). The studies with low, high, or unclear risk of bias and applicability concerns were summarized in Table 3, Figs. 4 and 5. Most studies showed a high risk of bias in the four domains. For example, the average cancer prevalence of included lesions was 39.5%, ranging from 6% to 64.7% (Supplementary Table 4 and Supplementary Fig. 1), which far exceeds the prevalence in screening and diagnostic settings^33^. This led to a high risk of bias in patient selection. Additionally, most study designs did not represent a complete US testing pathway applicable to clinical practice. For example, DL systems were used for image reading, but not integrated into clinical decisions, such as diagnosis, further tests, or follow-up. In contrast, the choice of patient management (e.g., biopsy, follow-up) to confirm disease status was based on the decision of the human readers rather than standalone or assistive DL systems. Meanwhile, for human readers, the testing pathway was also not applicable to clinical routines where they have access to patient’s clinical information as well as prior US images. The reference standards varied among the included 16 studies, of which 4 studies^17,22,25,28^ were at high risk of bias because the follow-up time of women with negative tests was <2 years, which is shorter than the recommended follow-up interval^33^ and therefore may underestimate the rate of missed cancers and overestimate diagnostic accuracy.

**Table 3:** Overview of concerns about risk of bias and applicability of studies using standalone DL systems or assistive DL systems.

Study	Risk of bias QUADAS-2				Applicability concerns QUADAS-2			Risk of bias QUADAS-C			
	P	I	R	FT	P	I	R	P	I	R	FT
*Standalone DL system*											
Kim 2021^18^	Low	High	High	High	High	High	Low	High	Low	Low	Low
Xiao 2019^19^	Unclear	Low	Low	Low	High	High	Low	High	Low	Low	Low
Cho 2018^20^	Low	Low	Low	Low	High	High	Low	Low	Low	Low	Low
Segni 2018^22^	Unclear	Low	High	High	High	High	High	High	Low	High	High
Lee 2022^24^	High	Low	Low	Low	High	High	Low	High	Low	Low	Low
Xia 2021^23^	Low	Low	Low	Low	High	High	Low	Low	Unclear	Low	Low
Choi 2019^25^	Low	Low	High	High	High	High	High	Low	Low	High	High
Nicosia 2022^26^	Low	Low	Low	Low	High	High	Low	Low	Low	Low	Low
Lai 2022^27^	Unclear	Low	Low	Low	High	High	Low	High	Low	Low	Low
Lee 2019^28^	High	Low	High	High	High	High	High	High	Low	High	High
Wei 2021^29^	High	Low	Low	Low	High	High	Low	High	Low	Low	Low
Wei 2022^30^	High	Low	Low	Low	High	High	Low	High	Low	Low	Low
Ciritsis 2019^31^	High	Low	Unclear	Unclear	High	High	Unclear	High	Low	High	High
Gu 2022^32^	Unclear	Low	Low	Low	High	High	Low	High	Low	Low	Low
*Assistive DL system*											
Park 2019^17^	Unclear	Low	High	High	High	High	High	High	Low	High	High
Kim 2021^18^	High	Low	Low	Low	High	High	Low	High	High	Low	Low
Cho 2018^20^	Low	Low	Low	Low	High	High	Low	Low	High	Low	Low
Wang 2021^21^	Low	Low	Low	Low	High	High	Low	Low	High	Low	Low
Xia 2021^23^	Low	Low	Low	Low	High	High	Low	High	Low	Low	Low
Lee 2022^24^	High	Low	Low	Low	High	High	Low	High	Low	Low	Low
Choi 2019^25^	Low	Low	High	High	High	High	High	Low	High	High	High
Lai 2022^27^	Unclear	Low	Low	Low	High	High	Low	High	Low	Low	Low
Lee 2019^28^	High	High	High	High	High	High	High	High	High	High	High
Wei 2021^29^	High	Low	Low	Low	High	High	Low	High	High	Low	Low
Wei 2022^30^	High	Low	Low	Low	High	High	Low	High	High	Low	Low
Gu 2022^32^	Unclear	Low	Low	Low	High	High	Low	High	Low	Low	Low

*P* patient selection, *I* index tests, *R* reference standard, *FT* flow and timing.

## Discussion

This review presents a comprehensive overview of diagnostic performance in breast US of DL systems, which serve as standalone roles or aids to human readers. We identified 16 studies that compared the test accuracy measures of a commercial or in-house DL system to that of human readers. Diagnostic test outcomes varied substantially among the included studies. While we cautiously inferred DL systems were more specific than average human readers, which might help decrease the false positives, no consensus of AUC, accuracy, and sensitivity was found either in standalone or assistive DL systems. Importantly, one of the main concerns of DL studies is better imaging sensitivity might come at the cost of increased false positives and vice versa. Critical performance metrics such as AUC, accuracy, sensitivity, specificity, true positive, false positive, false negative, and true negative should be taken into consideration together. However, not all included studies reported these diagnostic measures. Although most of the included studies (14/16) use FDA-approved DL systems, the clinical effects of DL systems as standalone or assistive roles have not been fully revealed yet due to the lack of generalizable reporting or good study design. Therefore, our systematic review disagrees with findings from various publications, some of which have claimed that DL systems (e.g., S-Detect) outperform humans^18,20,24^ and have a significant role in assisting human readers in distinguishing between benign and malignant breast masses^10,16^. It does not necessarily mean that the DL algorithm in breast US itself is unreliable. It contrarily provides the directions for future improvement for this promising technology.

Our review found high heterogeneity stemming from study designs, methods, targeted populations, diagnostic measures, and human readers’ experiences, which hinders the comparability of evidence across included studies. There was a wide variation in the number and pathological type of selected lesions. Thirteen studies evaluated fewer than 500 women while the outcomes of another three studies were based on many more participants. Promising results from small populations may not be applicable to larger populations. In addition, the malignant proportions far exceed the cancer prevalence in the real world, which inevitably overestimates the sensitivity. Importantly, most of the included studies originated in Asia, and mostly at a single site, which may affect the external validity of reported results. Furthermore, compared with Caucasian women, Asian women generally have denser breasts and younger ages of onset of breast cancer. Discrepancies in race and ethnicity make it difficult to extrapolate the positive findings among Asian participants to multi-race and multi-ethnic populations. Hence, multicenter studies from different countries that recruit participants from multiple races and ethnicities are required to achieve higher applicability of these studies. Additionally, the test cutoff values varied among studies with some using BIRADS-4a while some using BIRADS-4b as the threshold for classifying malignancies. In this regard, test bias could have been introduced. These studies also set various definitions of experienced or less experienced human readers, which might lead to contrary conclusions among some studies. Furthermore, the included studies have some variation in reference standards, including pathological confirmation and follow-up time (7–35 months). The methods for obtaining pathological results were also inconsistent, including histopathologic results from US-guided biopsy, vacuum-assisted excision, or open surgery. These discrepancies suggest that accuracy evaluations are not comparable among studies. Overall, the current evidence base is not of sufficient quality to support a broad clinical practice recommendation of DL systems in breast US.

Furthermore, compared to other medical imaging modalities, such as MRI, DL-assisted US shows intrinsic limitations, which hinders its clinical applicability. For example, US imaging is dependent on its operators, resulting in high intra- and inter-observer variability in image acquisition and interpretation. Moreover, unlike MRI images viewing the whole lesion range, still US images are obtained from parts of targeted organs, which may cause under-representation or over-exaggeration. Additionally, US technology has been evolving fast over the recent decades. Older ultrasonograms are generally of lower resolution and higher noise, while up-to-date images are of higher resolution and lower noise. Thus, DL models that are trained with older images may not be externally valid for images acquired by advanced devices. Methodological considerations are highly demanded for generalized conclusions from DL studies in US technology.

In this systematic review, we followed an established methodology and stringent inclusion criteria and tailored the quality assessment tools for included studies. Our emphasis on comparisons with the diagnostic performance of humans in clinical practice may explain why our conclusions are more cautious than many of the papers we reviewed herein. Importantly, according to previous studies and the current guidelines, internal validation where training and validation were performed based on the same dataset, such as cross-validation, tends to overestimate accuracy and has limited generalizability because of overfitting^33^. Hence, at the initial stage of literature identification, only studies using external validation of test sets were included. Therefore, our work can provide a purposeful insight into the role of DL in the US diagnosis of breast cancer. However, this systematic review excluded non-English publications, which might introduce selection bias. In addition, we were unable to calculate comprehensive diagnostic measures due to insufficient data where accuracy, true positive, false positive, true negative, false negative, and statistical difference (or raw data to calculate) were not reported.

To ensure reproducibility and generalizability of the results of this promising technology, we recommend developing standardized DL research guidelines for further investigations. Aligned study designs, agreed-upon benchmarking data sets, complete performance metrics, standard imaging protocols and reporting formats, consistent cutoff values and reference standards will help decrease the heterogeneity and bias. Furthermore, multicenter studies are highly demanded to determine the diagnostic accuracy of DL products. Prospective, randomized controlled trials that are applicable to clinical testing pathways are significantly important to examine DL’s role in a clinical environment. Also, we need to identify the DL products with the best performance in terms of accuracy, efficiency, availability, cost-effectiveness, and safety to improve clinical workflows. DL-based breast US diagnosis is still in its infancy, and considerable efforts are needed to realize its positive impacts on radiologists and patients.

## Methods

### Protocol and registration

This systematic review was conducted following the Preferred Reporting Items for Systematic Reviews and Meta-Analyses of Diagnostic Test Accuracy (PRISMA-DTA) statement^34^. Our review protocol was registered on the International Prospective Register of Systematic Reviews (PROSPERO: CRD42022349609).

### Literature search

Literature searches were conducted by two librarians (H.B. and J.B.) to identify relevant studies published in English from four databases: PubMed, Embase, Scopus, and Cochrane Library. The publication time of studies was set from inception to 18 January 2023. The literature search was performed based on five themes: breast cancer, US, AI, accuracy, and diagnostic. The search keywords and strategies are shown in Supplementary Tables 6 and 7.

### Study selection

Two reviewers (Q.D. and Z.X.) independently reviewed the titles and abstracts of all retrieved records for further identification according to the inclusion and exclusion criteria. Subsequently, the identified publications were screened by reviewing the full texts for final inclusion. Any discrepancies were resolved through discussion to reach a final consensus.

We applied rigorous inclusion and exclusion criteria to evaluate the integration of DL into clinical breast cancer diagnosis using the US. We included studies that focused on: (1) evaluating DL algorithms for breast cancer diagnosis using US; (2) assessing the test accuracy of DL algorithms for breast lesion diagnosis using US; and (3) utilizing histologically confirmed and/or follow-up reference standards. We excluded studies that: (1) did not compare the diagnostic performance of DL algorithms to that of human readers; (2) lacked external validation; (3) did not employ DL algorithms (e.g., utilizing traditional AI without binary classification or final decision); (4) solely focused on detecting specific cancer subtypes (e.g., ductal or lobular carcinoma) rather than overall diagnostic accuracy; (5) did not report diagnostic metrics beyond the receiver operating characteristic area under the curve (AUC); (6) involved participants under the age of 18; (7) included participants with implants, lactation, prior known breast cancer, or prior breast treatments such as surgery, radiation therapy, and chemotherapy; (8) enrolled male patients.

### Data extraction

Study characteristics and test accuracy outcomes were independently extracted by two reviewers (Q.D. and Z.X.) from all included studies. Any disagreements were resolved by discussion. Extracted study characteristics included study design, population, US device vendors, dataset characteristics (training/validation/testing set), descriptions of the DL algorithms, descriptions of the human readers, reference standards, and any other pertinent information. Test performance characteristics included accuracy, AUC, sensitivity, and specificity.

### Quality assessment

Two reviewers (Q.D. and Z.X.) independently assessed the quality of the selected studies using Quality for Assessment of Diagnostic Studies-2 (QUADAS-2) and QUADAS-C tools tailored to our review questions based on a breast US test pathway applicable to clinical settings (Supplementary Table 5). For risk of bias, patient selection, index tests, reference standards, flow, and timing were assessed, respectively. For applicability concerns, patient selection, index test, and reference standards were assessed. Any disagreements were resolved by discussion.

### Reporting summary

Further information on research design is available in the Nature Research Reporting Summary linked to this article.

### Supplementary information

Supporting information

Reporting summary

## Data Availability

All data generated and analyzed during this study are included in the article and its supplementary information files.
