# Supplementary material for: Diagnostic performance of deep learning in ultrasound diagnosis of breast cancer: a systematic review
Source: NPJ Precis Oncol. 2024 Jan 27;8:21. doi: 10.1038/s41698-024-00514-z (PMC10821881; doi:10.1038/s41698-024-00514-z)
Supplement: Supplementary file 1 — Supporting information [file 41698_2024_514_MOESM1_ESM.pdf]

Supporting information

Diagnostic accuracy of deep learning in ultrasound diagnosis of breast cancer: a systematic review

Qing Dan[1,2,#], Ziting Xu[1,#], Hannah Burrows[3], Jennifer Bissram[3], Jeffrey S. A. Stringer[2,*], Yingjia Li[1,*]

[1] Department of Ultrasound, Nanfang Hospital, Southern Medical University, Guangzhou 510515, China

[2] Global Women's Health, The University of North Carolina at Chapel Hill, Chapel Hill, NC, 27599, USA

[3] Health Sciences Library, The University of North Carolina at Chapel Hill, Chapel Hill, NC, 27599, USA

[#] Qing Dan and Ziting Xu contributed equally.

[*] Correspondence: lyjia@smu.edu.cn (Y.L.); jeffrey_stringer@med.unc.edu (J.S.).

Supplementary Table 1. Exclusion criteria in for domains, including population, intervention, comparison, and outcomes.

**Population**	
**Participants**	Female participants under 18 years old.
	Female with implants, lactation, or known breast cancer prior to ultrasound examination.
	Female who has undergone prior breast treatments, including surgery, radiation therapy, and chemotherapy.
	Studies involving male participants.
**Subtype images**	Studies using US images of subpopulations by test outcomes for DL algorithms training since they do not represent the population in screening or clinical settings. If commercial system, subimages (i.e., BIRADS-4), and single site data are allowed for reader study. If homemade system, subimages (i.e., BIRADS-4), and single site data are not allowed for models training.
**Prediction of cancer**	Studies using DL for the prediction of future cancer risk.
	Studies predicting cancer metastasis with DL systems.
**Intervention**	
**Not breast US**	Studies that investigated DL in mammography or using thyroid ultrasound DL algorithm for breast lesions classification, and other studies design without breast ultrasound.
**Not diagnostic DL**	Studies using traditional computer aided detection without classification or diagnosis.
**Internal validation**	For homemade DL systems, studies using internal validation (i.e., data from single site) where the validation dataset used to assess a model was also used to develop that model. Temporal validation which involves datasets only from single center should also be excluded.
	For commercial DL systems, data from single site is allowed for read study.

Comparison	
**Without involving human reader**	Studies that only develop DL systems rather than evaluate their diagnostic performance.
	Studies that don't compare the performance of DL algorithms and that of human readers.
**Without comparing diagnostic performance**	Studies that only compare the US images reading time, workflow efficiency, or biopsy rate of DL and human readers.
**Outcomes**	
**No relevant diagnostic metrics**	Studies only reporting diagnostic metrics like area under the curve (AUC), without specificity, sensitivity.

Supplementary Table 2. Main reasons for excluded references after full text review.

Number	Study	Reason
**Population**		
1	A novel approach with dual-sampling convolutional neural network for ultrasound image classification of breast tumors	Participants aged under 18
2	Application of computer-aided diagnosis in breast ultrasound interpretation: improvements in diagnostic performance according to reader experience	Participants had breast cancer history
3	Artificial intelligence system reduces false-positive findings in the interpretation of breast ultrasound exams	Participants aged under 18
4	Clinical value of radiomics and machine learning in breast ultrasound: a multicenter study for differential diagnosis of benign and malignant lesions	Participants aged under 18
5	Diagnostic performance of an artificial intelligence system in breast ultrasound	Male participants were included
6	Diagnostic value of breast lesions between deep learning-based computer-aided diagnosis system and experienced radiologists: comparison the performance between symptomatic and asymptomatic Patients	Eight participants underwent surgery
7	Feasibility of computer-assisted diagnosis for breast ultrasound: the results of the diagnostic performance of S-detect from a single center in China	Participants aged under 18
8	Reducing the number of unnecessary biopsies of US-BI-RADS 4a lesions through a deep learning method for residents-in-training: a cross-sectional study	Participants aged under 18
9	Should we Ignore, follow, or biopsy? Impact of artificial intelligence decision support on breast ultrasound lesion assessment	Participants aged under 18
10	Ultrasound-based deep learning in the establishment of a breast lesion risk stratification system: a multicenter study	Participants aged under 18
11	Dedicated computer-aided detection software for automated 3D breast ultrasound; an efficient tool for the radiologist in supplemental screening of women with dense breasts	Subimages
12	Machine learning-based diagnostic evaluation of shear-wave elastography in BI-RADS category 4 breast cancer screening: a multicenter, retrospective study	Subimages
13	Evaluating breast ultrasound S-detect image analysis for small focal breast lesions	Subimages

Intervention		
14	3-D Res-CapsNet convolutional neural network on automated breast ultrasound tumor diagnosis	Internal validation
15	A combined ultrasonic B-mode and color Doppler system for the classification of breast masses using neural network	Internal validation
16	A comparative study of multiple deep learning models based on multi-input resolution for breast ultrasound images	Internal validation
17	A generic deep learning framework to classify thyroid and breast lesions in ultrasound images	Internal validation
18	A machine learning ensemble based on radiomics to predict BI-RADS category and reduce the biopsy rate of ultrasound-detected suspicious breast masses	Internal validation
19	Application of deep learning to establish a diagnostic model of breast lesions using two-dimensional grayscale ultrasound imaging	Internal validation
20	Application of ultrasonic dual-mode artificially intelligent architecture in assisting radiologists with different diagnostic levels on breast masses classification	Internal validation
21	Breast classification in automated breast ultrasound using multiview convolutional neural network with transfer learning	Internal validation
22	Classification of breast cancer in ultrasound imaging using a generic deep learning analysis software: a pilot study	Internal validation
23	Classification of breast masses on ultrasound shear wave elastography using convolutional neural networks	Internal validation
24	Classification of breast ultrasound with human-rating BI-RADS scores using mined diagnostic patterns and optimized neuro-network	Internal validation
25	Classification of malignant tumors in breast ultrasound using a pretrained deep residual network model and support vector machine	Internal validation
26	Computer-aided analysis of ultrasound elasticity images for classification of benign and malignant breast masses	Internal validation
27	Computer-aided diagnosis of breast cancer in ultrasonography images by deep learning	Internal validation
28	Deep learning applied to two-dimensional color Doppler flow imaging ultrasound images significantly improves diagnostic performance in the classification of breast masses: a multicenter study	Internal validation

29	Diagnostic efficiency of the breast ultrasound computer-aided prediction model based on convolutional neural network in breast cancer	Internal validation
30	Discrimination of breast cancer based on ultrasound images and convolutional neural network	Internal validation
31	Diagnostic value of artificial intelligence automatic detection systems for breast BI-RADS 4 nodules	Internal validation
32	Distinction between benign and malignant breast masses at breast ultrasound using deep learning method with convolutional neural network	Internal validation
33	Fully automatic classification of automated breast ultrasound (ABUS) imaging according to BI-RADS using a deep convolutional neural network	Internal validation
34	Going beyond a first reader: a machine learning methodology for optimizing cost and performance in breast ultrasound diagnosis	Internal validation
35	Impact of radiomics on the breast ultrasound radiologist's clinical practice: from lumpologist to data wrangler	Internal validation
36	Improved Inception V3 method and its effect on radiologists' performance of tumor classification with automated breast ultrasound system	Internal validation
37	Machine learning to improve breast cancer diagnosis by multimodal ultrasound	Internal validation
38	One step further into the blackbox: a pilot study of how to build more confidence around an AI-based decision system of breast nodule assessment in 2D ultrasound	Internal validation
39	Palpable breast lump triage by minimally trained operators in Mexico using computer-assisted diagnosis and low-cost ultrasound	Internal validation
40	Performance of novel deep learning network with the incorporation of the automatic segmentation network for diagnosis of breast cancer in automated breast ultrasound	Internal validation
41	Prospective assessment of breast cancer risk from multimodal multiview ultrasound images via clinically applicable deep learning	Internal validation
42	Semi-supervised GAN-based radiomics model for data augmentation in breast ultrasound mass classification	Internal validation
43	Evaluating different combination methods to analyse ultrasound and shear wave elastography images automatically through discriminative convolutional neural network in breast cancer imaging	Internal validation

44	Establishment of a deep-learning system to diagnose BI-RADS4a or higher using breast ultrasound for clinical application	Internal validation
45	Artificial intelligence using open source BI-RADS data exemplifying potential future use	Not breast US
46	Enhancing performance of breast ultrasound in opportunistic screening women by a deep learning-based system: a multicenter prospective study	Not breast US
47	Intelligent breast tumor detection system with texture and contrast features	Not breast US
48	1000-case reader study of radiologists' performance in interpretation of automated breast volume scanner images with a computer-aided detection system	Not DL
49	Automated method for improving system performance of computer-aided diagnosis in breast ultrasound	Not DL
50	Breast US computer-aided diagnosis workstation: performance with a large clinical diagnostic population	Not DL
51	CAD algorithms for solid breast masses discrimination: evaluation of the accuracy and interobserver variability	Not DL
52	Computer aided classification system for breast ultrasound based on Breast Imaging Reporting and Data System (BI-RADS)	Not DL
53	Computer-aided classification of breast masses: performance and interobserver variability of expert radiologists versus residents	Not DL
54	Computer-aided diagnosis for surgical office-based breast ultrasound	Not DL
55	Computer-aided diagnosis system based on fuzzy logic for breast cancer categorization	Not DL
56	Computer-aided diagnosis of breast elastography	Not DL
57	Deep learning-based radiomics of B-mode ultrasonography and shear-wave elastography: improved performance in breast mass classification	Not DL
58	Evaluation of the accuracy of a computer-aided diagnosis (CAD) system in breast ultrasound according to the radiologist's experience	Not DL
59	Evaluation of the effect of computer-aided classification of benign and malignant lesions on reader performance in automated three-dimensional breast ultrasound	Not DL
60	Improved cancer detection in automated breast ultrasound by radiologists using computer aided detection	Not DL

61	Improved differential diagnosis of breast masses on ultrasonographic images with a computer-aided diagnosis scheme for determining histological classifications	Not DL
62	Interpretation time using a concurrent-read computer-aided detection system for automated breast ultrasound in breast cancer screening of women with dense breast tissue	Not DL
63	Machine learning models to improve the differentiation between benign and malignant breast lesions on ultrasound: a multicenter external validation study	Not DL
64	Management of breast lesions seen on US images: dual-model radiomics including shear-wave elastography may match performance of expert radiologists	Not DL
65	Multi-modality CADx: ROC study of the effect on radiologists' accuracy in characterizing breast masses on mammograms and 3D ultrasound images	Not DL
66	Novel computer-aided diagnosis algorithms on ultrasound image: effects on solid breast masses discrimination	Not DL
67	Performance and reading time of automated breast US with or without computer-aided detection	Not DL
68	Performance of computer-aided diagnosis in the interpretation of lesions on breast sonography	Not DL
69	Principal component regression-based contrast-enhanced ultrasound evaluation system for the management of BI-RADS US 4A breast masses: objective assistance for radiologists	Not DL
70	The feasibility of classifying breast masses using a computer-assisted diagnosis (CAD) system based on ultrasound elastography and BI-RADS lexicon	Not DL
71	The importance of multi-modal imaging and clinical information for humans and AI-based algorithms to classify breast masses (INSPiRED 003): an international, multicenter analysis	Not DL
72	Validation of radiologists' findings by computer-aided detection (CAD) software in breast cancer detection with automated 3D breast ultrasound: a concept study in implementation of artificial intelligence software	Not DL
73	Multi-modal artificial intelligence for the combination of automated 3D breast ultrasound and mammograms in a population of women with predominantly dense breasts	Not DL

74	An AI model of sonographer's evaluation+ S-Detect + elastography + clinical information improves the preoperative identification of benign and malignant breast masses	Not DL
75	Bi-Modal transfer learning for classifying breast cancers via combined B-mode and ultrasound strain imaging	Without involving human readers
**Outcomes**		
76	Impact of data presentation on physician performance utilizing artificial intelligence-based computer-aided diagnosis and decision support systems	Without diagnostic metrics
77	Impact of original and artificially improved artificial intelligence-based computer-aided diagnosis on breast US interpretation	Without diagnostic metrics
78	S-Detect characterization of focal solid breast lesions: a prospective analysis of inter-reader agreement for US BI-RADS descriptors	No relevant diagnostic accuracy
**Others**		
79	Classification method for samples that are easy to be confused in breast ultrasound images	Not English publication
80	Application of S-detect combined with virtual touch imaging quantification in ultrasound for diagnosis of breast mass	Not English publication

Supplementary Table 3. Additional information on US devices used in included studies.

Study	US vendor
Park 2019 [1]	RS80A (Samsung Medison, Seoul, Korea)
Kim 2021 [2]	RS85 Prestige (Samsung Medison, Seongnam, Korea)
Xiao 2019 [3]	RS80 Prestige (Samsung Medison, Seongnam, Korea)
Cho 2018 [4]	RS80A (Samsung Medison, Seoul, Korea)
Wang 2021 [5]	RS80A (Samsung Medison, Seoul, Korea)
Segni 2018 [6]	RS80A (Samsung Medison, Seoul, Korea)
Xia 2021 [7]	RS80A (Samsung Medison, Seoul, Korea)
Lee 2022 [8]	RS80A (Samsung Medison, Seoul, Korea)
Choi 2019 [9]	RS80A (Samsung Medison, Seoul, Korea)
Nicosia 2022 [10]	RS80A (Samsung Medison, Seoul, Korea)
Lai 2022 [11]	Philips iU22 (Philips Healthcare, Bothell, WA, USA),<br>Toshiba Aplio 500 (Toshiba Medical System Corporation, Tochigi, Japan)
Lee 2019 [12]	RS80A (Samsung Medison, Seoul, Korea)
Wei 2021 [13]	RS80A (Samsung Medison, Seoul, Korea)
Wei 2022 [14]	RS80A (Samsung Medison, Seoul, Korea)
Ciritsis 2019 [15]	Not reported
Gu 2022 [16]	Resona7, Resona7s, Resona7T, Resona8, Resona8T, and DC-80 (Shenzhen Mindray BioMedical Electronics, Shenzhen, China)

Supplementary Table 4. Additional cancer characteristics of included studies.

Study	Breast lesion (n)	Cancer prevalence n (%)	Cancer type n (%)	Tumor size
Park 2019 [1]	100	41 (41%)	Invasive ductal carcinoma 27 (27%)<br>Ductal carcinoma in situ 10 (10%)<br>Invasive lobular carcinoma 3 (3%)<br>Mucinous carcinoma 1 (1%)<br>Fibroadenoma or complex fibroadenoma 32 (32%)<br>Fibrocystic changes 7 (7%)<br>Intraductal papilloma 5 (5%)<br>Mammary duct ectasia 4 (4%)<br>Benign phyllodes tumor 3 (3%)<br>Nodular adenosis 2 (2%)<br>Radial scar 1 (1%)<br>Suture granuloma 1 (1%)	Overall: 14±7 mm (range, 4–39 mm)<br>Malignant: 14±8 mm (range, 4–39 mm)<br>Benign: 12±7mm (range, 4–37mm)

			Sclerosing adenosis 1 (1%)<br>No diagnostic abnormality 1 (1%)<br>Fibroadipose tissue 1 (1%)<br>Fibroadenomatoid hyperplasia 1 (1%)	
Kim 2021 [2]	156	10 (6.4%)	Invasive ductal carcinomas 8 (5.1%)<br>Ductal carcinomas in situ 2 (1.3%)<br>Fibroadenomas 58 (37%)<br>Fibrocystic changes 21 (13.5%)<br>Intraductal papillomas 14 (9%)<br>Fibroadenomatoid changes 13 (8.3%)<br>Sclerosing adenoses 13 (8.3%)<br>Stromal fibroses 5 (3.2%)<br>Atypical ductal hyperplasias 4 (2.6%)<br>4 usual ductal hyperplasias (2.6%)<br>Duct ectasias 4 (2.6%)<br>Benign phyllodes tumors 3 (1.9%)	11±5 mm (range, 3–34 mm)

			Atypical ductal hyperplasias involving intraductal papillomas 2 (1.3%)<br><br>Fat necroses 2 (1.3%)<br><br>Nodular adenosis 1 (0.64%)<br><br>Cholesterol granuloma 1 (0.64%)<br><br>Adenomyoepithelioma 1 (0.64%)	
Xiao 2019 [3]	448	218 (48.7%)	Not reported	Not reported
Cho 2018 [4]	119	54 (45.4%)	No reported	16.9±10.7 mm (range, 4–60 mm)
Wang 2021 [5]	173	95 (54.9 %)	Invasive ductal carcinoma 59 (34.1%)<br><br>Ductal carcinoma in situ 11 (6.36%)<br><br>Solid papillary carcinoma 4 (2.31%)<br><br>Invasive lobular carcinoma 2 (1.16%)<br><br>Mucinous carcinoma 2 (1.16%)<br><br>Benign proliferative disease 45 (26.01%)	16 mm± 9mm (range, 4–46 mm)

			Fibroadenoma 23 (13.29%)<br>Intraductal papilloma 9 (5.20%)<br>Inflammation 8 (4.62%)<br>Others 10 (5.78%)	
Segni 2018 [6]	68	44 (64.7%)	Infiltrating ductal carcinomas 37 (54.4%)<br>Ductal carcinomas in situ 3 (4.4%)<br>Infiltrating lobular carcinomas 3 (4.4%)<br>Granular cell tumor 1 (1.5%)<br>Fibroadenomas 12 (17.6%)<br>Phyllodes tumor 1 (1.5%)<br>Hamartomas 2 (2.9%)<br>Sclerosing adenosis and/or fibrocystic mastopathy 7 (10.3%)<br>Abscesses 2 (2.9%)	Range, 10–48 mm

Xia 2021[7]	40	24 (60%)	Invasive ductal carcinoma 17 (42.5%)<br>Mucinous carcinoma 1 (2.5%)<br>Papillary carcinoma 1 (2.5%)<br>Intraductal carcinoma 3 (7.5%)<br>Invasive lobular carcinoma 1 (2.5%)<br>Intraductal carcinoma in situ 1 (2.5%)<br>Mammary gland disease 4 (10%)<br>Fibroadenoma 8 (20%)<br>papilloma 2 (5%)<br>Other benign tumors 2 (5%)	Not reported
Lee 2022[8]	492	200 (40.7%)	Invasive ductal carcinoma 171 (34.76%)<br>Ductal carcinoma in situ 14 (2.85%)<br>Invasive lobular carcinoma 11 (2.34%)<br>Tubular carcinoma 4 (0.81%)<br>Fibroadenoma 99 (20.12%)<br>Fibroadenomatoid hyperplasia 22 (4.47%)	14.2±7.5 mm (range, 4–48 mm)

			Intraductal papilloma 17 (3.46%)<br>Stromal fibroses 14 (2.85%)<br>Fibrocystic changes 13 (2.64%)<br>Others 44 (8.94%)<br>Stable for more than 2 years 83 (16.9%)	
Choi 2019 [9]	253	80 (31.62%)	Invasive ductal carcinoma 67 (26.48%)<br>Ductal carcinoma in situ 9 (3.56%)<br>Invasive lobular carcinoma 3 (1.19%)<br>Invasive papillary carcinoma 1 (0.40%)<br>Fibroadenoma 43 (17.00%)<br>Fibrocystic changes 6 (2.37%)<br>Intraductal papilloma 6 (2.37%)<br>Phyllodes tumor 5 (1.98%)<br>Stromal fibroses 2 (0.79%)<br>Fibroadenomatoid mastopathy 2 (0.79%)<br>Adenosis 2 (0.79%)	11 mm (IQR, 8–17 mm)<br>Benign 10 mm (7–13 mm)<br>Malignant 17 mm (12–25 mm)

			Lobular carcinoma in situ 1 (0.00%)<br>Cyst 1 (0.40%)<br>Others 105 (41.50%)	
Nicosia 2022 [10]	256	142 (55.5%)	Invasive ductal carcinoma 107 (41.8%)<br>Invasive lobular carcinoma 10 (3.9%)<br>Cribriform 4 (1.6%)<br>Apocrine carcinoma 2 (0.8%)<br>Mucinous carcinoma 3 (1.2%)<br>Metaplastic carcinoma 1 (0.4%)<br>Low grade intraductal carcinoma 5 (1.9%)<br>Intermediate intraductal carcinoma 5 (1.9%)<br>High grade intraductal carcinoma 2 (0.8%)<br>Neuroendocrine intraductal carcinoma 2 (0.8%)<br>Diffuse large B cell lymphoma 1 (0.4%)<br>Fibroadenoma 63 (24.6%)<br>Fibrocystic disease 13 (5.1%)	18.6 mm (SD = 9.1 mm)

			Adenosis 15 (5.8%)<br>Chronic inflammation 14 (5.5%)<br>Hamartoma 1 (0.4%)<br>Atypical lobular hyperplasia 1 (0.4%)<br>Intraductal papilloma 5 (1.9%)<br>Gynecomastia 2 (0.8%)	
Lai 2022 [11]	172	65 (37.79%)	Ductal carcinoma in situ 7 (4.07%)<br>Intraductal carcinoma 52 (30.23%)<br>Infiltrating ductal carcinomas 3 (1.74%)<br>Others 3 (1.74%)<br>Benign 107 (62.2%)	Not reported
Lee 2019 [12]	500	68 (13.6%)	Not reported	1.19±0.78 cm

Wei 2021 [13]	266	69 (25.94%)	Invasive carcinomas 59 (22.12%)<br>Ductal carcinoma in situ 6 (2.26%)<br>Metastatic squamous cell carcinoma 2 (0.75%)<br>Mucinous carcinoma 2 (0.75%)<br>Benign 197 (74.06%)	14.8±9.2 mm (range, 5–54 mm)
Wei 2022 [14]	901	326 (35.82%)	Invasive ductal carcinoma 276 (30.63%)<br>Invasive lobular carcinoma 7 (0.78%)<br>Intraductal carcinoma 31 (3.44%)<br>Medullary carcinoma 3 (0.33%)<br>Mucinous carcinoma 5 (0.55%)<br>Invasive papillary carcinoma 2 (0.22%)<br>Leiomyosarcoma 1 (0.11%)<br>Paget's disease 1 (0.11%)<br>Proliferative disease 352 (39.06%)<br>Fibroadenoma 179 (19.87%)<br>Intraductal papilloma 13 (1.44%)	17.2±9.2 mm

			Chronic inflammation 14 (1.55%)<br><br>Phyllodes tumor 4 (0.44%)<br><br>Cyst 4 (0.44%)<br><br>Abscess 2 (0.22%)<br><br>Granular cell tumor 1 (0.11%)<br><br>Others 9 (1%)						
Ciritsis 2019 [15]	Not reported	Not reported	Not reported				Not reported		
Gu 2022 [16]	5012	1792 (35.75%)	Training<br><br>Invasive carcinoma 1288 (31.05%)<br><br>Ductal carcinoma in situ 110 (2.65%)<br><br>Solid papillary carcinoma 10 (0.24%)<br><br>Encapsulated papillary carcinoma 6 (0.15%)	Internal<br><br>Invasive carcinoma 143 (30.70%)<br><br>Ductal carcinoma in situ 14 (3.01%)<br><br>Solid papillary carcinoma 1 (0.21%)<br><br>Encapsulated papillary carcinoma 0 (0.00)	External<br><br>Invasive carcinoma 171 (43.08%)<br><br>Ductal carcinoma in situ 22 (5.54%)<br><br>Solid papillary carcinoma 5 (1.26%)<br><br>Encapsulated papillary carcinoma 5 (1.26%)	Overall<br><br>Invasive carcinoma 1602 (31.96%)<br><br>Ductal carcinoma in situ 146 (2.91%)<br><br>Solid papillary carcinoma 16 (0.32%)<br><br>Encapsulated papillary carcinoma 11 (0.22%)	Training<br><br>18.4±9.9 (3–74.6) mm	Internal<br><br>17.6±9.4 (4–68) mm	External<br><br>20.5±10.8 (3.5–70) mm

			Lobular tumor, malignant/borderline 3 (0.07%)<br><br>Other malignant lesions 8 (0.19%)	Lobular tumor, malignant/borderline 1 (0.21%)<br><br>Other malignant lesions 2 (0.42%)	Lobular tumor, malignant/borderline 2 (0.50%)<br><br>Other malignant lesions 1 (0.25%)	Lobular tumor, malignant/borderline 6 (0.12%)<br><br>Other malignant lesions 11 (0.22%)			

Supplementary Table 5. Quality assessment of included studies according to QUADAS-2 and QUADAS-C tools, adapted from previous report [17] .

**Domain 1: Patient Selection**		
**Single test accuracy (QUADAS-2): risk of bias**		
Signaling questions	1.1 Was a consecutive or random sample of patients enrolled?	**Yes** - RCTs and cohort studies (prospective or retrospective).<br>**No** - Other studies.<br>**Unclear** - If not stated.
	1.2 Was a case-control design avoided?	**Yes** - If any of the following statements<br>(1) Each patient receiving all of the index tests (fully paired design);<br>(2) Random allocation of patients to one of the index tests (randomized design).<br>**No** - Other studies.<br>**Unclear** - If not stated.
	1.3 Did the study avoid inappropriate exclusions?	**Yes** - If inappropriate exclusions were avoided.<br>It generated a consecutive or truly random allocation sequence of female patients and US images.<br>**No** - If any of the following statements<br>(1) Exclusion of more than 10% [17] of the samples for any reason, for example retrospective studies with missing data (i.e., lost to follow up);<br>(2) Exclusion of types of women/images, i.e., BIRADS category;<br>(3) Exclusion based on outcomes, i.e., cancer types, interval cancers, recall decision.<br>**Unclear** - If not clearly reported.
	1.4 Were the women and US images included in the study independent of those used to train the AI models?	**For test set studies,**<br>**Yes** - External geographical validation (test set was sample from a different center; can be in another country or the same country).<br>**No** - Any internal validation (i.e., split sample, cross-validation) or temporal validation.<br>**Unclear** - No details stated about the training set and testing set.<br><br>**For prospective studies in a clinical context,**<br>**Yes** - If the study was located at different center (s) providing US images used to train and test the DL model (geographical validation).

		**No** - If there was any overlap between model development dataset and model assessment dataset.<br>**Unclear** - If not stated.
Risk of bias	1.5 Could the selection of patients have introduced bias?	**Low** - If questions 1.1 to 1.4 were answered 'yes'.<br>**High** - If at least one question was answered 'no'; if question 1.2 was answered 'no', strongly consider 'high risk of bias'.<br>**Unclear** - Only be used when insufficient data were reported.
Concerns regarding applicability	Are there concerns that the included patients do not match the review question?	**Low** - If 'no' for all the following statements.<br>**High** - If 'yes' for any of the following statements.<br>**Unclear** - If no details were provided.<br>(1) Not a consecutive or random sample of women enrolled;<br>(2) Enriched sample/cancer prevalence doesn't match clinical context (>3%) [17];<br>(3) US images only subset, i.e., recalled cases, BIRADS-4 categorized images;<br>(4) US images of enrolled women not representative of women in worldwide population (ethnicity, age).
**Comparative accuracy (QUADAS-C): risk of bias**		
Signaling questions	C1.1 Was the risk of bias for each index test judged 'low' for this domain?	**Yes** - If the risk of bias judgment for single test accuracy (question 1.5 in QUADAS-2) was 'low' for each index test.<br>**No** - Otherwise.
	C1.2 Was a fully paired or randomized design used?	**Yes** - If one of the following<br>(1) Each patient receiving all of the index tests (fully paired design);<br>(2) random allocation of patients to one of the index tests (randomized design).<br>**No** - If not a fully paired or randomized design.<br>**Unclear** - If not stated.
	C1.3 Was the allocation sequence random?<br><br>(This question only applicable to randomized designs.)	**Yes** - If the study generated a truly random allocation sequence, i.e., computer-generated random numbers.<br>**No** - Otherwise.<br>**Unclear** - If not stated.<br>**Not applicable**
	C1.4 Was the allocation sequence concealed until	**Yes** - If the study used appropriate methods to conceal allocation, such as central randomization schemes and opaque sealed envelopes.

	patients were enrolled and assigned to index tests?<br><br>(This question only applicable to randomized designs.)	**No** - Otherwise.<br>**Unclear** - If not stated.<br>**Not applicable**
Risk of bias	C1.5 Could the selection of patients have introduced bias?	**Low** - If questions C1.1 to C1.4 were answered 'yes'.<br>**High** - If at least one question was answered 'no'; if question C1.2 was answered 'no', strongly consider 'high risk of bias'.<br>**Unclear** - Only be used when insufficient data are reported.

Domain 2: Index tests		
**Single test accuracy (QUADAS-2): risk of bias**		
Signaling questions	2.1 Were the index test results interpreted without knowledge of the results of the reference standard?	**Yes** - Require clear statement of blinding, or clear temporal relationships where the human read occurred before the reference standard.<br>**No** - Otherwise.<br>**Unclear** - If not stated.
	2.2 If a threshold was used, was it prespecified?	**Yes** - If any of the following statements (1) Using a commercially available DL system which gave a yes/no result, or threshold clearly pre-specified in methods; (2) For systems giving a risk score and study explicitly states the pre-specified threshold.<br>**No** - Otherwise.<br>**Unclear** - If not stated.
Risk of bias	2.3 Could the conduct or interpretation of the index test have introduced bias?	**Low** - If questions 2.1 to 2.2 were answered 'yes'.<br>**High** - If at least one question was answered 'no'.<br>**Unclear** - Only be used when insufficient data were reported.

Concerns regarding applicability	Are there concerns that the index test, its conduct or its interpretation differ from the review question?	**Low** - If 'no' for all the following statements.<br>**High** - If 'yes' for any of the following statements.<br>**Unclear** - If no details were provided.<br>(1) DL system not yet commercially available, i.e., in-house systems;<br>(2) Study did not use a pre-specified threshold for DL system;<br>(3) Not a complete testing pathway applicable to clinical practice. For example, DL model for images reading, but not integrated into clinical decisions, such as diagnosis, further test, or follow up;<br>(4) Human comparator was not a complete testing pathway applicable to clinical practice where there has human double reading with arbitration at clinical threshold;<br>(5) DL model/human reader had no access to prior US images.

Comparative accuracy (QUADAS-C): risk of bias		
Signaling questions	C2.1 Was the risk of bias for each index test judged ‘low’ for this domain?	**Yes** - If the risk of bias judgment for single test accuracy (question 2.3 in QUADAS-2) was ‘low’ for each index test.<br>**No** - Otherwise.
	C2.2 Were the index test results interpreted without knowledge of the results of the other index test(s)?<br><br>(This question only applicable if patients received multiple index tests with fully or partially paired designs.)	**Yes** -<br>For standalone DL system, if the interpretation of DL and human reader were blind to each other.<br><br>For assistive DL system, if the interpretation of [DL] group and [human reader + DL] group were blind to each other. For example, there is a wash out period between human reader reading images and making final decision after knowing the diagnosis from DL.<br>**No** - Otherwise.<br>**Unclear** - If not stated.<br>**Not applicable**
	C2.3 Was undergoing one index test unlikely to affect the performance of the other index test(s)?<br><br>(This question only applicable if patients received multiple index tests with fully or partially paired designs.)	**Yes** - If test outcomes of DL model cannot subsequently influence or interfere with the results of human reader, and vice versa. Of note, for assistive DL system (human + DL), to compare human and [human + DL], human readers in two reading scenarios should be different, or there should be a washout time in two reading scenarios if human reader are same.<br>**No** - Otherwise.<br>**Unclear** - If not stated.<br>**Not applicable**
	C2.4 Were the index tests conducted and interpreted without advantaging one of the tests?	**Yes** - If there were no differences between the index tests that may unfairly benefit one of the tests.<br>**No** - Otherwise.<br>**Unclear** - If not stated.
Risk of bias	C2.5 Could the conduct or interpretation of the index tests have introduced bias in the comparison?	**Low** - If questions C2.1 to C2.4 were answered ‘yes’.<br>**High** - If at least one question was answered ‘no’.<br>**Unclear** - Only be used when insufficient data were reported.

Domain 3: Reference Standard		
**Single test accuracy (QUADAS-2): risk of bias**		
Signaling questions	3.1 Is the reference standard likely to correctly classify the target condition?	**Yes** - If any of the following statements.<br>(1) Histopathology results;<br>(2) With at least 2 years follow up to exclude interval cancers.<br>**No -** Otherwise.<br>**Unclear** - If not stated.
	3.2 Were the reference standard results interpreted without knowledge of the results of the index test?	**Yes**<br>**No**<br>**Unclear**
Risk of bias	3.3 Could the reference standard, its conduct, or its interpretation have introduced bias?	**Low** - If questions 3.1 to 3.2 were answered 'yes'.<br>**High** - If at least one question was answered 'no'.<br>**Unclear** - Only be used when insufficient data were reported.
Concerns regarding applicability	Are there concerns that the target condition as defined by the reference standard does not match the review question?	**Low** - If 'no' for all the following statements.<br>**High** - If 'yes' for any of the following statements.<br>**Unclear** - If no details were provided.<br>(1) Length of examination rounds (if included women underwent more than one US examination for follow up) less than 2 years for follow-up;<br>(2) Classification not by biopsy/follow-up.
**Comparative accuracy (QUADAS-C): risk of bias**		
Signaling questions	C3.1 Was the risk of bias for each index test judged 'low' for this domain?	**Yes** - If the risk of bias judgment for single test accuracy (question 3.3 in QUADAS-2) was 'low' for each index test.<br>**No** - Otherwise.
	C3.2 Did the reference standard avoid incorporating any of the index tests?	**Yes** - If none of the index tests were part of the reference standard. Note that this issue is different from blinding (signaling question 3.2 in QUADAS-2).<br>**No** - Otherwise.<br>**Unclear** - If not stated.
Risk of bias	C3.3 Could the reference standard, its conduct, or its interpretation have introduced bias in the comparison?	**Low** - If signaling questions C3.1 and C3.2 were answered 'yes'.<br>**High** - If at least one question was answered 'no'.

		**Unclear** - Only be used when insufficient data are reported.

Domain 4: Flow and Timing		
**Single test accuracy (QUADAS-2): risk of bias**		
Signaling questions	4.1 Was there an appropriate interval between index tests and reference standard?	**Yes** - If appropriate time interval applied to exclude disease progression.<br>**No -** Otherwise.<br>**Unclear** - If not stated.
	4.2 Did all patients receive a reference standard?	**No -** If any of the following statements<br>(1) There was significant (>10%) loss to follow up for reference standards of interval cancers or subsequent examination results.<br>(2) If any women who should have received a biopsy or follow-up tests after index test positive results did not receive one or results were unavailable.<br>**Yes -** Otherwise.<br>**Unclear** - If not stated.
	4.3 Did all patients receive the same reference standard?	**Yes** - If all patients receive the same reference standard.<br>**No -** Otherwise.<br>**Unclear** - If not stated.
	4.4 Were all patients included in the analysis?	**Yes**<br>**No -** If there were any exclusions after the point of selecting the cohort.<br>**Unclear** - If not stated.
Risk of bias	4.5 Could the patient flow have introduced bias?	**Low** - If questions 4.1 to 4.2 were answered 'yes'.<br>**High** - If at least one question was answered 'no'.<br>**Unclear** - Only be used when insufficient data were reported.
**Comparative accuracy (QUADAS-C): risk of bias**		

Signaling questions	C4.1 Was the risk of bias for each index test judged 'low' for this domain?	**Yes** - If the risk of bias judgment for single test accuracy (question 4.5 in QUADAS-2) was 'low' for each index test.<br>**No** - Otherwise.
	C4.2 Was there an appropriate interval between the index tests?	**Yes** - For prospective study, if appropriate time interval applied to exclude disease progression. For retrospective study, it doesn't matter whether there's time interval between DL model and human reader.<br>**No** - Otherwise.<br>**Unclear** - If not stated.
	C4.3 Was the same reference standard used for all index tests?	**Yes** - If any of the following statements<br>(1) A single reference standard was used in all patients;<br>(2) In RTC study where multiple reference standards were used (i.e., either pathology or follow-up), these reference standards were the same for DL model and human reader.<br>**No** - Otherwise.<br>**Unclear** - If not stated.
	C4.4 Are the proportions and reasons for missing data similar across index tests? (Missing data occurs if test results are unavailable, invalid, inconclusive, or if patients are excluded from the analysis.)	**Yes** - If there was no missing data, or if the proportion and reasons for missing data are similar for DL model and human reader.<br>**No** - Otherwise.<br>**Unclear** - If not stated.
Risk of bias	C4.5 Could the patient flow have introduced bias in the comparison?	**Low** - If signaling questions C4.1 to C4.4 were answered 'yes'.<br>**High** - If at least one question was answered 'no'.<br>**Unclear** - Only be used when insufficient data are reported.

Supplementary Table 6. Summary of initial search strategies from inception to 25 August, 2022.

PubMed		
Search #	Query	Results
1 – breast cancer	(Breast-Neoplasms[mesh] OR breast-neoplasms[tiab] OR breast-neoplasm[tiab] OR breast-tumor[tiab] OR breast-cancer[tiab] OR Breast-Tumors[tiab] OR Mammary-Cancer[tiab] OR Mammary-Cancers[tiab] OR Malignant-Neoplasm-of-Breast[tiab] OR Breast-Malignant-Neoplasm[tiab] OR Breast-Malignant-Neoplasms[tiab] OR Malignant-Tumor-of-Breast[tiab] OR Breast-Malignant-Tumor[tiab] OR Breast-Malignant-Tumors[tiab] OR Cancer-of-Breast[tiab] OR Cancer-of-the-Breast[tiab] OR Human-Mammary-Carcinomas[tiab] OR Human-Mammary-Carcinoma[tiab] OR Human-Mammary-Neoplasms[tiab] OR Breast-Carcinoma[tiab] OR Breast-Carcinomas[tiab] OR breast-lesion[tiab] OR breast-lesions[tiab] OR Carcinoma,-Ductal,-Breast[mesh] OR Carcinoma,-Lobular[mesh] OR lobular-carcinoma[tiab] OR lobular-carcinomas[tiab] OR Mammary-Ductal-Carcinomas[tiab] OR Mammary-Ductal-Carcinoma[tiab])	431,510
2 – ultrasound	(Ultrasonography,-Mammary[mesh] OR Mammary-Ultrasonography[tiab] OR Breast-Ultrasonography[tiab] OR Breast-Ultrasonographies[tiab] OR Ultrasonography[mesh] OR ultrasound[tiab] OR ultrasounds[tiab] OR sonography[tiab] OR ultrasonic-imaging[tiab] OR radiologist[tiab] OR radiologists[mesh] OR radiologists[tiab] OR human-reader[tiab] OR human-readers[tiab])	680,501
3 – AI	(Artificial-intelligence[mesh] OR Artificial-intelligence[tiab] OR Algorithms[mesh] OR Algorithms[tiab] OR Deep-learning[mesh] OR Deep-learning[tiab] OR Neural-Networks,-Computer[mesh] OR Computational-Intelligence[tiab] OR Machine-Intelligence[tiab] OR Computer-Vision-Systems[tiab] OR Computer-Vision-System[tiab] OR Computer-Neural-Network[tiab] OR Computer-Neural-Networks[tiab] OR Neural-Network-Model[tiab] OR Neural-Network-Models[tiab] OR Computational-Neural-Networks[tiab] OR Computational-Neural-Network[tiab] OR Diagnosis,-Computer-Assisted[mesh] OR Computer-Assisted-Diagnosis[tiab] OR Computer-Assisted-Diagnosis[tiab] OR Computer-Assisted-Diagnoses[tiab] OR machine-learning[tiab])	565,008
4 – accuracy	(Diagnostic-errors[mesh] OR Diagnostic-errors[tiab] OR Diagnostic-Error[tiab] OR Misdiagnosis[tiab] OR Misdiagnoses[tiab] OR Reproducibility-of-Results[mesh] OR Reproducibility-of-Results[tiab] OR Reproducibility-of-Findings[tiab] OR Reproducibility-Of-Result[tiab] OR	2,345,870

	Reproducibility-of-Finding[tiab] OR Finding-Reproducibility[tiab] OR Reliability-of-Results[tiab] OR Reliability-of-Result[tiab] OR Result-Reliability[tiab] OR Validity-of-Results[tiab] OR Validity-of-Result[tiab] OR Result-Validity[tiab] OR Reliability-and-Validity[tiab] OR Validity-and-Reliability[tiab] OR Test-Retest-Reliability[tiab] OR Accuracy[tiab] OR Observer-variation[mesh] OR Observer-variation[tiab] OR Observer-Variations[tiab] OR Observer-Bias[tiab] OR Interobserver-Variation[tiab] OR Interobserver-Variations[tiab] OR Inter-Observer-Variation[tiab] OR Inter-Observer-Variations[tiab] OR Interobserver-Variability[tiab] OR Interobserver-Variabilities[tiab] OR Inter-Observer-Variability[tiab] OR Inter-Observer-Variabilities[tiab] OR Intraobserver-Variation[tiab] OR Intraobserver-Variations[tiab] OR Intra-Observer-Variation[tiab] OR Intra-Observer-Variations[tiab] OR Intraobserver-Variability[tiab] OR Intraobserver-Variabilities[tiab] OR Intra-Observer-Variability[tiab] OR Intra-Observer-Variabilities[tiab] OR sensitivity-and-specificity[mesh] OR sensitivity-and-specificity[tiab] OR sensitivity[tiab] OR specificity[tiab] OR False-positive[tiab] OR False-negative[tiab] OR Missed-diagnosis[mesh] OR Missed-diagnosis[tiab] OR missed-diagnoses[tiab] OR Test-performance[tiab] OR Diagnostic-accuracy[tiab])	
5 – diagnostic	(Early-detection-of-cancer[mesh] OR Diagnosis[mesh] OR Detection[tiab] OR Cancer-Screening[tiab] OR Early-Diagnosis-of-Cancer[tiab] OR Cancer-Early-Diagnosis[tiab] OR Diagnoses[tiab] OR Diagnose[tiab] OR Diagnoses-and-Examinations[tiab] OR Examinations-and-Diagnoses[tiab] OR Diagnoses-and-Examination[tiab] OR Examination-and-Diagnoses[tiab] OR Postmortem-Diagnosis[tiab] OR Postmortem-Diagnoses[tiab] OR Antemortem-Diagnosis[tiab] OR Antemortem-Diagnoses[tiab] OR Diagnostic-imaging[mesh] OR Medical-imaging[tiab] OR diagnostic-imaging[tiab] OR Early-detection-of-cancer[tiab] OR Diagnosis[tiab])	10,679,891
6 – COMBINED	#1 AND #2 AND #3 AND #4 AND #5	1,830

Embase		
Search #	Query	Results
1 – breast cancer	(breast-cancer/exp OR breast-cancer:ab,ti OR breast-cancers:ab,ti OR Breast-Neoplasms:ab,ti OR breast-neoplasm:ab,ti OR breast-tumor/exp OR breast-tumor:ab,ti OR Breast-Tumors:ab,ti OR Mammary-Cancer:ab,ti OR Mammary-Cancers:ab,ti OR Malignant-Neoplasm-of-Breast:ab,ti OR Breast-Malignant-Neoplasm:ab,ti OR Breast-Malignant-Neoplasms:ab,ti OR Malignant-Tumor-of-Breast:ab,ti OR	693,569

	Breast-Malignant-Tumor:ab,ti OR Breast-Malignant-Tumors:ab,ti OR Cancer-of-Breast:ab,ti OR Cancer-of-the-Breast:ab,ti OR Human-Mammary-Carcinomas:ab,ti OR Human-Mammary-Carcinoma:ab,ti OR Human-Mammary-Neoplasm:ab,ti OR Human-Mammary-Neoplasms:ab,ti OR Breast-Carcinoma/exp OR Breast-Carcinoma:ab,ti OR Breast-Carcinomas:ab,ti OR breast-lesion/exp OR breast-lesion:ab,ti OR breast-lesions:ab,ti OR breast-ductal-carcinoma/exp OR breast-ductal-carcinoma:ab,ti OR breast-ductal-carcinomas:ab,ti OR lobular-carcinoma/exp OR lobular-carcinoma:ab,ti OR lobular-carcinomas:ab,ti OR Mammary-Ductal-Carcinomas:ab,ti OR Mammary-Ductal-Carcinoma:ab,ti)	
2 – ultrasound	(echomammography/exp OR echomammography:ab,ti OR echomammographies:ab,ti OR Mammary-Ultrasonography:ab,ti OR Mammary-Ultrasonographies:ab,ti OR Breast-Ultrasonography:ab,ti OR Breast-Ultrasonographies:ab,ti OR ultrasonography:ab,ti OR echography:ab,ti OR ultrasound/exp OR ultrasound:ab,ti OR ultrasounds:ab,ti OR sonography:ab,ti OR ultrasonic-imaging:ab,ti OR radiologist/exp OR radiologist:ab,ti OR radiologists:ab,ti OR human-reader:ab,ti OR human-readers:ab,ti)	748,192
3 – AI	(Artificial-intelligence/exp OR Artificial-intelligence:ab,ti OR Algorithm/exp OR Algorithm:ab,ti OR Algorithms:ab,ti OR Deep-learning/exp OR Deep-learning:ab,ti OR artificial-neural-network/exp OR artificial-neural-network:ab,ti OR artificial-neural-networks:ab,ti OR Computational-Intelligence:ab,ti OR Machine-Intelligence:ab,ti OR Computer-Vision:ab,ti OR Computer-Neural-Network:ab,ti OR Computer-Neural-Networks:ab,ti OR Neural-Network-Model:ab,ti OR Neural-Network-Models:ab,ti OR Computational-Neural-Networks:ab,ti OR Computational-Neural-Network:ab,ti OR Computer-Assisted-Diagnosis/exp OR Computer-Assisted-Diagnosis:ab,ti OR Computer-Assisted-Diagnoses:ab,ti OR machine-learning/exp OR machine-learning:ab,ti)	2,137,197
4 – accuracy	(Diagnostic-errors:ab,ti OR Diagnostic-error:ab,ti OR Diagnostic-Error/exp OR Misdiagnosis:ab,ti OR Misdiagnoses:ab,ti OR Reproducibility/exp OR Reproducibility-of-Results:ab,ti OR Reproducibility-of-Findings:ab,ti OR Reproducibility-Of-Result:ab,ti OR Reproducibility-of-Finding:ab,ti OR Finding-Reproducibilities:ab,ti OR Finding-Reproducibility:ab,ti OR Reliability-of-Results:ab,ti OR Reliability/exp OR Result-Reliabilities:ab,ti OR Result-Reliability:ab,ti OR Validity-of-Results:ab,ti OR Validity/exp OR Result-Validities:ab,ti OR Result-Validity:ab,ti OR Reliability-and-Validity:ab,ti OR Validity-and-Reliability:ab,ti OR Test-Retest-Reliability:ab,ti OR Accuracy/exp OR Accuracy:ab,ti OR Observer-variation/exp OR Observer-variation:ab,ti OR Observer-Variations:ab,ti OR Observer-Bias/exp OR Observer-Bias:ab,ti OR Observer-Biases:ab,ti OR Interobserver-Variation:ab,ti OR Interobserver-Variations:ab,ti	2,977,098

	OR Inter-Observer-Variation:ab,ti OR Inter-Observer-Variations:ab,ti OR Interobserver-Variability:ab,ti OR Interobserver-Variabilities:ab,ti OR Inter-Observer-Variability:ab,ti OR Inter-Observer-Variabilities:ab,ti OR Intraobserver-Variation:ab,ti OR Intraobserver-Variations:ab,ti OR Intra-Observer-Variation:ab,ti OR Intra-Observer-Variations:ab,ti OR Intraobserver-Variability:ab,ti OR Intraobserver-Variabilities:ab,ti OR Intra-Observer-Variability:ab,ti OR Intra-Observer-Variabilities:ab,ti OR sensitivity-and-specificity/exp OR sensitivity-and-specificity:ab,ti OR sensitivity:ab,ti OR specificity:ab,ti OR false-positive-result/exp OR False-positive*:ab,ti OR false-negative-result/exp OR False-negative*:ab,ti OR Missed-diagnosis/exp OR Missed-diagnosis:ab,ti OR missed-diagnoses:ab,ti OR task-performance/exp OR Test-performance*:ab,ti OR Diagnostic-accuracy/exp OR Diagnostic-accuracy:ab,ti OR diagnostic-accuracies:ab,ti)	
5 – diagnostic	(Early-detection-of-cancer:ab,ti OR early-cancer-diagnosis/exp OR early-cancer-diagnosis:ab,ti OR Diagnosis/exp OR diagnosis:ab,ti OR cancer-diagnosis/exp OR Detection:ab,ti OR Cancer-Screening/exp OR Cancer-Screening:ab,ti OR cancer-screenings:ab,ti OR Early-Diagnosis/exp OR Cancer-Early-Diagnosis:ab,ti OR Diagnoses:ab,ti OR Diagnose:ab,ti OR Diagnoses-and-Examinations:ab,ti OR Examinations-and-Diagnoses:ab,ti OR Diagnoses-and-Examination:ab,ti OR Examination-and-Diagnoses:ab,ti OR Diagnostic-imaging/exp OR diagnostic-imag*:ab,ti OR Medical-imag*:ab,ti)	9,713,064
6 – COMBINED	#1 AND #2 AND #3 AND #4 AND #5	3,521
7 – w/o abstracts	#6 AND ([article]/lim OR [article in press]/lim OR [data papers]/lim OR [review]/lim OR [short survey]/lim OR [preprint]/lim)	3,008

Scopus		
Search #	Query	Results
1 – breast cancer	TITLE-ABS(breast-neoplasms OR breast-neoplasm OR breast-tumor OR breast-cancer OR Breast-Tumors OR Mammary-Cancer OR Mammary-Cancers OR Malignant-Neoplasm-of-Breast OR Breast-Malignant-Neoplasm OR Breast-Malignant-Neoplasms OR Malignant-Tumor-of-Breast OR Breast-Malignant-Tumor OR Breast-Malignant-Tumors OR Cancer-of-Breast OR Cancer-of-the-Breast OR Human-Mammary-Carcinomas OR Human-Mammary-Carcinoma OR Human-Mammary-Neoplasm OR Human-Mammary-Neoplasms OR Breast-Carcinoma OR Breast-Carcinomas OR breast-lesion OR breast-lesions OR lobular-carcinoma OR lobular-carcinomas	421,821

	OR Mammary-Ductal-Carcinomas OR Mammary-Ductal-Carcinoma)	
2 – ultrasound	TITLE-ABS(Mammary-Ultrasonography OR Mammary-Ultrasonographies OR Breast-Ultrasonography OR Breast-Ultrasonographies OR ultrasound OR ultrasounds OR sonography OR ultrasonic-imaging OR radiologist OR radiologists OR human-reader OR human-readers)	517,000
3 – AI	TITLE-ABS(Artificial-intelligence OR Algorithms OR Deep-learning OR Computational-Intelligence OR Machine-Intelligence OR Computer-Vision-Systems OR Computer-Vision-System OR Computer-Neural-Network OR Computer-Neural-Networks OR Neural-Network-Model OR Neural-Network-Models OR Computational-Neural-Networks OR Computational-Neural-Network OR Computer-Assisted-Diagnosis OR Computer-Assisted-Diagnosis OR Computer-Assisted-Diagnoses OR machine-learning)	3,528,295
4 – accuracy	TITLE-ABS(Diagnostic-errors OR Diagnostic-Error OR Misdiagnosis OR Misdiagnoses OR Reproducibility-of-Results OR Reproducibility-of-Findings OR Reproducibility-Of-Result OR Reproducibility-of-Finding OR Finding-Reproducibilities OR Finding-Reproducibility OR Reliability-of-Results OR Reliability-of-Result OR Result-Reliabilities OR Result-Reliability OR Validity-of-Results OR Validity-of-Result OR Result-Validities OR Result-Validity OR Reliability-and-Validity OR Validity-and-Reliability OR Test-Retest-Reliability OR Accuracy OR Observer-variation OR Observer-Variations OR Observer-Bias OR Interobserver-Variation OR Interobserver-Variations OR Inter-Observer-Variation OR Inter-Observer-Variations OR Interobserver-Variability OR Interobserver-Variabilities OR Inter-Observer-Variability OR Inter-Observer-Variabilities OR Intraobserver-Variation OR Intraobserver-Variations OR Intra-Observer-Variation OR Intra-Observer-Variations OR Intraobserver-Variability OR Intraobserver-Variabilities OR Intra-Observer-Variability OR Intra-Observer-Variabilities OR sensitivity-and-specificity OR sensitivity OR specificity OR False-positive OR False-negative OR Missed-diagnosis OR missed-diagnoses OR Test-performance OR Diagnostic-accuracy)	4,281,157
5 – diagnostic	TITLE-ABS(Detection OR Cancer-Screening OR Early-Diagnosis-of-Cancer OR Cancer-Early-Diagnosis OR Diagnoses OR Diagnose OR Diagnoses-and-Examinations OR Examinations-and-Diagnoses OR Diagnoses-and-Examination OR Examination-and-Diagnoses OR Postmortem-Diagnosis OR Postmortem-Diagnoses OR Antemortem-Diagnosis OR Antemortem-Diagnoses OR Medical-imaging OR diagnostic-imaging OR Early-detection-of-cancer OR Diagnosis)	4,809,408
6 – COMBINED	#1 AND #2 AND #3 AND #4 AND #5	946

7 – w/o abstracts	#6 AND (LIMIT-TO (DOCTYPE , "ar") OR LIMIT-TO(DOCTYPE , "re"))	617

Cochrane Library		
Search #	Query	Results
1 – breast cancer	((breast-neoplasms OR breast-neoplasm OR breast-tumor OR breast-cancer OR Breast-Tumors OR Mammary-Cancer OR Mammary-Cancers OR Malignant-Neoplasm-of-Breast OR Breast-Malignant-Neoplasm OR Breast-Malignant-Neoplasms OR Malignant-Tumor-of-Breast OR Breast-Malignant-Tumor OR Breast-Malignant-Tumors OR Cancer-of-Breast OR Cancer-of-the-Breast OR Human-Mammary-Carcinomas OR Human-Mammary-Carcinoma OR Human-Mammary-Neoplasm OR Human-Mammary-Neoplasms OR Breast-Carcinoma OR Breast-Carcinomas OR breast-lesion OR breast-lesions OR lobular-carcinoma OR lobular-carcinomas OR Mammary-Ductal-Carcinomas OR Mammary-Ductal-Carcinoma)):ti,ab,kw	40,644
2 – ultrasound	((Mammary-Ultrasonography OR Mammary-Ultrasonographies OR Breast-Ultrasonography OR Breast-Ultrasonographies OR ultrasound OR ultrasounds OR sonography OR ultrasonic-imaging OR radiologist OR radiologists OR human-reader OR human-readers)):ti,ab,kw	40,280
3 – AI	((Artificial-intelligence OR Algorithms OR Deep-learning OR Computational-Intelligence OR Machine-Intelligence OR Computer-Vision-Systems OR Computer-Vision-System OR Computer-Neural-Network OR Computer-Neural-Networks OR Neural-Network-Model OR Neural-Network-Models OR Computational-Neural-Networks OR Computational-Neural-Network OR Computer-Assisted-Diagnosis OR Computer-Assisted-Diagnosis OR Computer-Assisted-Diagnoses OR machine-learning)):ti,ab,kw	17,731
4 – accuracy	((Diagnostic-errors OR Diagnostic-Error OR Misdiagnosis OR Misdiagnoses OR Reproducibility OR Reliability OR Accuracy OR sensitivity OR specificity OR False-positive OR False-negative OR Missed-diagnosis OR missed-diagnoses OR Test-performance OR Diagnostic-accuracy)):ti,ab,kw	104,971
5 – diagnostic	((Detection OR Cancer-Screening OR Diagnoses OR Diagnose OR Diagnosis OR Medical-imaging OR diagnostic-imaging OR Early-detection-of-cancer)):ti,ab,kw	214,782
6 – COMBINED	#1 AND #2 AND #3 AND #4 AND #5	31

Total before duplicates removed: 5486
Total after duplicates removed: 3847

Supplementary Table 7. Summary of updated search after initial search from 25 August, 2022 to 18 January, 2023.

PubMed		
Search #	Query	Results
1 – breast cancer	(Breast-Neoplasms[mesh] OR breast-neoplasms[tiab] OR breast-neoplasm[tiab] OR breast-tumor[tiab] OR breast-cancer[tiab] OR Breast-Tumors[tiab] OR Mammary-Cancer[tiab] OR Mammary-Cancers[tiab] OR Malignant-Neoplasm-of-Breast[tiab] OR Breast-Malignant-Neoplasm[tiab] OR Breast-Malignant-Neoplasms[tiab] OR Malignant-Tumor-of-Breast[tiab] OR Breast-Malignant-Tumor[tiab] OR Breast-Malignant-Tumors[tiab] OR Cancer-of-Breast[tiab] OR Cancer-of-the-Breast[tiab] OR Human-Mammary-Carcinomas[tiab] OR Human-Mammary-Carcinoma[tiab] OR Human-Mammary-Neoplasms[tiab] OR Breast-Carcinoma[tiab] OR Breast-Carcinomas[tiab] OR breast-lesion[tiab] OR breast-lesions[tiab] OR Carcinoma,-Ductal,-Breast[mesh] OR Carcinoma,-Lobular[mesh] OR lobular-carcinoma[tiab] OR lobular-carcinomas[tiab] OR Mammary-Ductal-Carcinomas[tiab] OR Mammary-Ductal-Carcinoma[tiab])	440,670
2 – ultrasound	(Ultrasonography,-Mammary[mesh] OR Mammary-Ultrasonography[tiab] OR Breast-Ultrasonography[tiab] OR Breast-Ultrasonographies[tiab] OR Ultrasonography[mesh] OR ultrasound[tiab] OR ultrasounds[tiab] OR sonography[tiab] OR ultrasonic-imaging[tiab] OR radiologist[tiab] OR radiologists[mesh] OR radiologists[tiab] OR human-reader[tiab] OR human-readers[tiab])	692,395
3 – AI	(Artificial-intelligence[mesh] OR Artificial-intelligence[tiab] OR Algorithms[mesh] OR Algorithms[tiab] OR Deep-learning[mesh] OR Deep-learning[tiab] OR Neural-Networks,-Computer[mesh] OR Computational-Intelligence[tiab] OR Machine-Intelligence[tiab] OR Computer-Vision-Systems[tiab] OR Computer-Vision-System[tiab] OR Computer-Neural-Network[tiab] OR Computer-Neural-Networks[tiab] OR Neural-Network-Model[tiab] OR Neural-Network-Models[tiab] OR Computational-Neural-Networks[tiab] OR Computational-Neural-Network[tiab] OR Diagnosis,-Computer-Assisted[mesh] OR Computer-Assisted-Diagnosis[tiab] OR Computer-Assisted-Diagnosis[tiab] OR Computer-Assisted-Diagnoses[tiab] OR machine-learning[tiab])	590,177
4 – accuracy	(Diagnostic-errors[mesh] OR Diagnostic-errors[tiab] OR Diagnostic-Error[tiab] OR Misdiagnosis[tiab] OR Misdiagnoses[tiab] OR Reproducibility-of-Results[mesh] OR Reproducibility-of-Results[tiab] OR Reproducibility-of-Findings[tiab] OR Reproducibility-Of-Result[tiab] OR	2,399,130

	Reproducibility-of-Finding[tiab] OR Finding-Reproducibility[tiab] OR Reliability-of-Results[tiab] OR Reliability-of-Result[tiab] OR Result-Reliability[tiab] OR Validity-of-Results[tiab] OR Validity-of-Result[tiab] OR Result-Validity[tiab] OR Reliability-and-Validity[tiab] OR Validity-and-Reliability[tiab] OR Test-Retest-Reliability[tiab] OR Accuracy[tiab] OR Observer-variation[mesh] OR Observer-variation[tiab] OR Observer-Variations[tiab] OR Observer-Bias[tiab] OR Interobserver-Variation[tiab] OR Interobserver-Variations[tiab] OR Inter-Observer-Variation[tiab] OR Inter-Observer-Variations[tiab] OR Interobserver-Variability[tiab] OR Interobserver-Variabilities[tiab] OR Inter-Observer-Variability[tiab] OR Inter-Observer-Variabilities[tiab] OR Intraobserver-Variation[tiab] OR Intraobserver-Variations[tiab] OR Intra-Observer-Variation[tiab] OR Intra-Observer-Variations[tiab] OR Intraobserver-Variability[tiab] OR Intraobserver-Variabilities[tiab] OR Intra-Observer-Variability[tiab] OR Intra-Observer-Variabilities[tiab] OR sensitivity-and-specificity[mesh] OR sensitivity-and-specificity[tiab] OR sensitivity[tiab] OR specificity[tiab] OR False-positive[tiab] OR False-negative[tiab] OR Missed-diagnosis[mesh] OR Missed-diagnosis[tiab] OR missed-diagnoses[tiab] OR Test-performance[tiab] OR Diagnostic-accuracy[tiab])	
5 – diagnostic	(Early-detection-of-cancer[mesh] OR Diagnosis[mesh] OR Detection[tiab] OR Cancer-Screening[tiab] OR Early-Diagnosis-of-Cancer[tiab] OR Cancer-Early-Diagnosis[tiab] OR Diagnoses[tiab] OR Diagnose[tiab] OR Diagnoses-and-Examinations[tiab] OR Examinations-and-Diagnoses[tiab] OR Diagnoses-and-Examination[tiab] OR Examination-and-Diagnoses[tiab] OR Postmortem-Diagnosis[tiab] OR Postmortem-Diagnoses[tiab] OR Antemortem-Diagnosis[tiab] OR Antemortem-Diagnoses[tiab] OR Diagnostic-imaging[mesh] OR Medical-imaging[tiab] OR diagnostic-imaging[tiab] OR Early-detection-of-cancer[tiab] OR Diagnosis[tiab])	10,826,507
6 – COMBINED	#1 AND #2 AND #3 AND #4 AND #5	1,882
7 – AFTER 8/25/22	#6 AND 2022/08/25:3000/12/12[crdt]	44

Embase		
Search #	Query	Results
1 – breast cancer	(breast-cancer/exp OR breast-cancer:ab,ti OR breast-cancers:ab,ti OR Breast-Neoplasms:ab,ti OR breast-neoplasm:ab,ti OR breast-tumor/exp OR breast-tumor:ab,ti OR Breast-Tumors:ab,ti OR Mammary-Cancer:ab,ti OR Mammary-Cancers:ab,ti OR Malignant-Neoplasm-of-Breast:ab,ti OR Breast-Malignant-Neoplasm:ab,ti OR Breast-Malignant-Neoplasms:ab,ti OR Malignant-Tumor-of-Breast:ab,ti OR Breast-Malignant-Tumor:ab,ti OR Breast-Malignant-Tumors:ab,ti OR Cancer-of-Breast:ab,ti OR Cancer-of-the-Breast:ab,ti OR Human-Mammary-Carcinomas:ab,ti OR Human-Mammary-Carcinoma:ab,ti OR Human-Mammary-Neoplasm:ab,ti OR Human-Mammary-Neoplasms:ab,ti OR Breast-Carcinoma/exp OR Breast-Carcinoma:ab,ti OR Breast-Carcinomas:ab,ti OR breast-lesion/exp OR breast-lesion:ab,ti OR breast-lesions:ab,ti OR breast-ductal-carcinoma/exp OR breast-ductal-carcinoma:ab,ti OR breast-ductal-carcinomas:ab,ti OR lobular-carcinoma/exp OR lobular-carcinoma:ab,ti OR lobular-carcinomas:ab,ti OR Mammary-Ductal-Carcinomas:ab,ti OR Mammary-Ductal-Carcinoma:ab,ti)	710,002
2 – ultrasound	(echomammography/exp OR echomammography:ab,ti OR echomammographies:ab,ti OR Mammary-Ultrasonography:ab,ti OR Mammary-Ultrasonographies:ab,ti OR Breast-Ultrasonography:ab,ti OR Breast-Ultrasonographies:ab,ti OR ultrasonography:ab,ti OR echography:ab,ti OR ultrasound/exp OR ultrasound:ab,ti OR ultrasounds:ab,ti OR sonography:ab,ti OR ultrasonic-imaging:ab,ti OR radiologist/exp OR radiologist:ab,ti OR radiologists:ab,ti OR human-reader:ab,ti OR human-readers:ab,ti)	768,192
3 – AI	(Artificial-intelligence/exp OR Artificial-intelligence:ab,ti OR Algorithm/exp OR Algorithm:ab,ti OR Algorithms:ab,ti OR Deep-learning/exp OR Deep-learning:ab,ti OR artificial-neural-network/exp OR artificial-neural-network:ab,ti OR artificial-neural-networks:ab,ti OR Computational-Intelligence:ab,ti OR Machine-Intelligence:ab,ti OR Computer-Vision:ab,ti OR Computer-Neural-Network:ab,ti OR Computer-Neural-Networks:ab,ti OR Neural-Network-Model:ab,ti OR Neural-Network-Models:ab,ti OR Computational-Neural-Networks:ab,ti OR Computational-Neural-Network:ab,ti OR Computer-Assisted-Diagnosis/exp OR Computer-Assisted-Diagnosis:ab,ti OR Computer-Assisted-Diagnoses:ab,ti OR machine-learning/exp OR machine-learning:ab,ti)	2,225,222
4 – accuracy	(Diagnostic-errors:ab,ti OR Diagnostic-error:ab,ti OR Diagnostic-Error/exp OR Misdiagnosis:ab,ti OR Misdiagnoses:ab,ti OR Reproducibility/exp OR Reproducibility-of-Results:ab,ti OR Reproducibility-of-Findings:ab,ti OR	3,054,507

	Reproducibility-Of-Result:ab,ti OR Reproducibility-of-Finding:ab,ti OR Finding-Reproducibilities:ab,ti OR Finding-Reproducibility:ab,ti OR Reliability-of-Results:ab,ti OR Reliability/exp OR Result-Reliabilities:ab,ti OR Result-Reliability:ab,ti OR Validity-of-Results:ab,ti OR Validity/exp OR Result-Validities:ab,ti OR Result-Validity:ab,ti OR Reliability-and-Validity:ab,ti OR Validity-and-Reliability:ab,ti OR Test-Retest-Reliability:ab,ti OR Accuracy/exp OR Accuracy:ab,ti OR Observer-variation/exp OR Observer-variation:ab,ti OR Observer-Variations:ab,ti OR Observer-Bias/exp OR Observer-Bias:ab,ti OR Observer-Biases:ab,ti OR Interobserver-Variation:ab,ti OR Interobserver-Variations:ab,ti OR Inter-Observer-Variation:ab,ti OR Inter-Observer-Variations:ab,ti OR Interobserver-Variability:ab,ti OR Interobserver-Variabilities:ab,ti OR Inter-Observer-Variability:ab,ti OR Inter-Observer-Variabilities:ab,ti OR Intraobserver-Variation:ab,ti OR Intraobserver-Variations:ab,ti OR Intra-Observer-Variation:ab,ti OR Intra-Observer-Variations:ab,ti OR Intraobserver-Variability:ab,ti OR Intraobserver-Variabilities:ab,ti OR Intra-Observer-Variability:ab,ti OR Intra-Observer-Variabilities:ab,ti OR sensitivity-and-specificity/exp OR sensitivity-and-specificity:ab,ti OR sensitivity:ab,ti OR specificity:ab,ti OR false-positive-result/exp OR False-positive*:ab,ti OR false-negative-result/exp OR False-negative*:ab,ti OR Missed-diagnosis/exp OR Missed-diagnosis:ab,ti OR missed-diagnoses:ab,ti OR task-performance/exp OR Test-performance*:ab,ti OR Diagnostic-accuracy/exp OR Diagnostic-accuracy:ab,ti OR diagnostic-accuracies:ab,ti)	
5 – diagnostic	(Early-detection-of-cancer:ab,ti OR early-cancer-diagnosis/exp OR early-cancer-diagnosis:ab,ti OR Diagnosis/exp OR diagnosis:ab,ti OR cancer-diagnosis/exp OR Detection:ab,ti OR Cancer-Screening/exp OR Cancer-Screening:ab,ti OR cancer-screenings:ab,ti OR Early-Diagnosis/exp OR Cancer-Early-Diagnosis:ab,ti OR Diagnoses:ab,ti OR Diagnose:ab,ti OR Diagnoses-and-Examinations:ab,ti OR Examinations-and-Diagnoses:ab,ti OR Diagnoses-and-Examination:ab,ti OR Examination-and-Diagnoses:ab,ti OR Diagnostic-imaging/exp OR diagnostic-imag*:ab,ti OR Medical-imag*:ab,ti)	9,937,209
6 – COMBINED	#1 AND #2 AND #3 AND #4 AND #5	3,694
7 – w/o abstracts	#6 AND ([article]/lim OR [article in press]/lim OR [data papers]/lim OR [review]/lim OR [short survey]/lim OR [preprint]/lim)	3,153
8 – Added to Embase after 8/25/2022	#7 [25-08-2022]/sd NOT [02-02-2023]/sd	218
9 – Published in 2022 or 2023	#8 AND (2022:py OR 2023:py)	192

Scopus		
Search #	Query	Results
1 – breast cancer	TITLE-ABS(breast-neoplasms OR breast-neoplasm OR breast-tumor OR breast-cancer OR Breast-Tumors OR Mammary-Cancer OR Mammary-Cancers OR Malignant-Neoplasm-of-Breast OR Breast-Malignant-Neoplasm OR Breast-Malignant-Neoplasms OR Malignant-Tumor-of-Breast OR Breast-Malignant-Tumor OR Breast-Malignant-Tumors OR Cancer-of-Breast OR Cancer-of-the-Breast OR Human-Mammary-Carcinomas OR Human-Mammary-Carcinoma OR Human-Mammary-Neoplasm OR Human-Mammary-Neoplasms OR Breast-Carcinoma OR Breast-Carcinomas OR breast-lesion OR breast-lesions OR lobular-carcinoma OR lobular-carcinomas OR Mammary-Ductal-Carcinomas OR Mammary-Ductal-Carcinoma)	433,261
2 – ultrasound	TITLE-ABS(Mammary-Ultrasonography OR Mammary-Ultrasonographies OR Breast-Ultrasonography OR Breast-Ultrasonographies OR ultrasound OR ultrasounds OR sonography OR ultrasonic-imaging OR radiologist OR radiologists OR human-reader OR human-readers)	531,541
3 – AI	TITLE-ABS(Artificial-intelligence OR Algorithms OR Deep-learning OR Computational-Intelligence OR Machine-Intelligence OR Computer-Vision-Systems OR Computer-Vision-System OR Computer-Neural-Network OR Computer-Neural-Networks OR Neural-Network-Model OR Neural-Network-Models OR Computational-Neural-Networks OR Computational-Neural-Network OR Computer-Assisted-Diagnosis OR Computer-Assisted-Diagnosis OR Computer-Assisted-Diagnoses OR machine-learning)	3,677,001
4 – accuracy	TITLE-ABS(Diagnostic-errors OR Diagnostic-Error OR Misdiagnosis OR Misdiagnoses OR Reproducibility-of-Results OR Reproducibility-of-Findings OR Reproducibility-Of-Result OR Reproducibility-of-Finding OR Finding-Reproducibilities OR Finding-Reproducibility OR Reliability-of-Results OR Reliability-of-Result OR Result-Reliabilities OR Result-Reliability OR Validity-of-Results OR Validity-of-Result OR Result-Validities OR Result-Validity OR Reliability-and-Validity OR Validity-and-Reliability OR Test-Retest-Reliability OR Accuracy OR Observer-variation OR Observer-Variations OR Observer-Bias OR Interobserver-Variation OR Interobserver-Variations OR Inter-Observer-Variation OR Inter-Observer-Variations OR Interobserver-Variability OR Interobserver-Variabilities OR Inter-Observer-Variability OR Inter-Observer-Variabilities OR Intraobserver-Variation OR Intraobserver-Variations OR Intra-Observer-Variation OR Intra-Observer-Variations OR Intraobserver-Variability OR	4,428,734

	Intraobserver-Variabilities OR Intra-Observer-Variability OR Intra-Observer-Variabilities OR sensitivity-and-specificity OR sensitivity OR specificity OR False-positive OR False-negative OR Missed-diagnosis OR missed-diagnoses OR Test-performance OR Diagnostic-accuracy)	
5 – diagnostic	TITLE-ABS(Detection OR Cancer-Screening OR Early-Diagnosis-of-Cancer OR Cancer-Early-Diagnosis OR Diagnoses OR Diagnose OR Diagnoses-and-Examinations OR Examinations-and-Diagnoses OR Diagnoses-and-Examination OR Examination-and-Diagnoses OR Postmortem-Diagnosis OR Postmortem-Diagnoses OR Antemortem-Diagnosis OR Antemortem-Diagnoses OR Medical-imaging OR diagnostic-imaging OR Early-detection-of-cancer OR Diagnosis)	4,952,971
6 – COMBINED	#1 AND #2 AND #3 AND #4 AND #5	1,021
7 – w/o abstracts	#6 AND (LIMIT-TO (DOCTYPE , "ar") OR LIMIT-TO(DOCTYPE , "re"))	238
8 – Publication year 2022 or 2023	#8 AND (LIMIT-TO (PUBYEAR , 2023) OR LIMIT-TO (PUBYEAR , 2022)	190

Cochrane Library		
Search #	Query	Results
1 – breast cancer	((breast-neoplasms OR breast-neoplasm OR breast-tumor OR breast-cancer OR Breast-Tumors OR Mammary-Cancer OR Mammary-Cancers OR Malignant-Neoplasm-of-Breast OR Breast-Malignant-Neoplasm OR Breast-Malignant-Neoplasms OR Malignant-Tumor-of-Breast OR Breast-Malignant-Tumor OR Breast-Malignant-Tumors OR Cancer-of-Breast OR Cancer-of-the-Breast OR Human-Mammary-Carcinomas OR Human-Mammary-Carcinoma OR Human-Mammary-Neoplasm OR Human-Mammary-Neoplasms OR Breast-Carcinoma OR Breast-Carcinomas OR breast-lesion OR breast-lesions OR lobular-carcinoma OR lobular-carcinomas OR Mammary-Ductal-Carcinomas OR Mammary-Ductal-Carcinoma)):ti,ab,kw	42,031
2 – ultrasound	((Mammary-Ultrasonography OR Mammary-Ultrasonographies OR Breast-Ultrasonography OR Breast-Ultrasonographies OR ultrasound OR ultrasounds OR sonography OR ultrasonic-imaging OR radiologist OR radiologists OR human-reader OR human-readers)):ti,ab,kw	43,461
3 – AI	((Artificial-intelligence OR Algorithms OR Deep-learning OR Computational-Intelligence OR Machine-Intelligence OR Computer-Vision-Systems OR Computer-Vision-System OR Computer-Neural-Network OR Computer-Neural-Networks OR Neural-Network-Model OR Neural-Network-Models OR Computational-Neural-Networks OR Computational-Neural-Network OR Computer-Assisted-Diagnosis OR Computer-	10,169

	Assisted-Diagnosis OR Computer-Assisted-Diagnoses OR machine-learning)):ti,ab,kw	
4 – accuracy	((Diagnostic-errors OR Diagnostic-Error OR Misdiagnosis OR Misdiagnoses OR Reproducibility OR Reliability OR Accuracy OR sensitivity OR specificity OR False-positive OR False-negative OR Missed-diagnosis OR missed-diagnoses OR Test-performance OR Diagnostic-accuracy)):ti,ab,kw	108,829
5 – diagnostic	((Detection OR Cancer-Screening OR Diagnoses OR Diagnose OR Diagnosis OR Medical-imaging OR diagnostic-imaging OR Early-detection-of-cancer)):ti,ab,kw	223,195
6 – COMBINED	#1 AND #2 AND #3 AND #4 AND #5	33
7 – Pub date after 8/25/2022	#6 with Cochrane Library publication date from Aug 2022 to Jul 2023	3

Total before duplicates removed: 429
Total after duplicates removed: 345

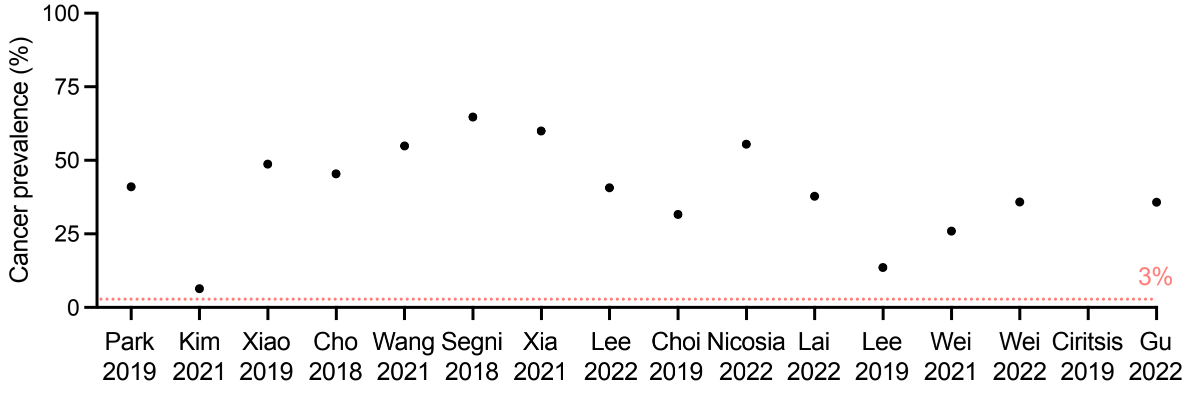

Supplementary Figure 1. Cancer prevalence in the included studies. A cancer prevalence of 3% that occurs in clinical practice is used for reference [17].

Reference

1. Park, H. J. *et al.* A computer-aided diagnosis system using artificial intelligence for the diagnosis and characterization of breast masses on ultrasound: Added value for the inexperienced breast radiologist. *Medicine (Baltimore)* **98**, e14146 (2019).
2. Kim, M. Y., Kim, S.-Y., Kim, Y. S., Kim, E. S. & Chang, J. M. Added value of deep learning-based computer-aided diagnosis and shear wave elastography to b-mode ultrasound for evaluation of breast masses detected by screening ultrasound. *Medicine (Baltimore)* **100**, e26823 (2021).
3. Xiao, M. *et al.* An investigation of the classification accuracy of a deep learning framework-based computer-aided diagnosis system in different pathological types of breast lesions. *J Thorac Dis* **11**, 5023–5031 (2019).
4. Cho, E., Kim, E.-K., Song, M. K. & Yoon, J. H. Application of Computer-Aided Diagnosis on Breast Ultrasonography: Evaluation of Diagnostic Performances and Agreement of Radiologists According to Different Levels of Experience. *J Ultrasound Med* **37**, 209–216 (2018).
5. Wang, X.-Y., Cui, L.-G., Feng, J. & Chen, W. Artificial intelligence for breast ultrasound: An adjunct tool to reduce excessive lesion biopsy. *Eur J Radiol* **138**, 109624 (2021).
6. Di Segni, M. *et al.* Automated classification of focal breast lesions according to S-detect: validation and role as a clinical and teaching tool. *J Ultrasound* **21**, 105–118 (2018).
7. Xia, Q. *et al.* Differential diagnosis of breast cancer assisted by S-Detect artificial intelligence system. *Math Biosci Eng* **18**, 3680–3689 (2021).
8. Lee, S. E. *et al.* Differing benefits of artificial intelligence-based computer-aided diagnosis for breast US according to workflow and experience level. *Ultrasonography* **41**, 718–727 (2022).

9. Choi, J. S. *et al.* Effect of a Deep Learning Framework-Based Computer-Aided Diagnosis System on the Diagnostic Performance of Radiologists in Differentiating between Malignant and Benign Masses on Breast Ultrasonography. *Korean J Radiol* **20**, 749 (2019).

10. Nicosia, L. *et al.* Evaluation of computer-aided diagnosis in breast ultrasonography: Improvement in diagnostic performance of inexperienced radiologists. *Clin Imaging* **82**, 150–155 (2022).

11. Lai, Y.-C. *et al.* Evaluation of physician performance using a concurrent-read artificial intelligence system to support breast ultrasound interpretation. *The Breast* **65**, 124–135 (2022).

12. Lee, J., Kim, S., Kang, B. J., Kim, S. H. & Park, G. E. Evaluation of the effect of computer aided diagnosis system on breast ultrasound for inexperienced radiologists in describing and determining breast lesions. *Med Ultrason* **21**, 239 (2019).

13. Wei, Q. *et al.* The Added Value of a Computer-Aided Diagnosis System in Differential Diagnosis of Breast Lesions by Radiologists With Different Experience. *J of Ultrasound Medicine* **41**, 1355–1363 (2022).

14. Wei, Q. *et al.* The diagnostic performance of ultrasound computer-aided diagnosis system for distinguishing breast masses: a prospective multicenter study. *Eur Radiol* **32**, 4046–4055 (2022).

15. Ciritsis, A. *et al.* Automatic classification of ultrasound breast lesions using a deep convolutional neural network mimicking human decision-making. *Eur Radiol* **29**, 5458–5468 (2019).

16. Gu, Y. *et al.* Deep learning based on ultrasound images assists breast lesion diagnosis in China: a multicenter diagnostic study. *Insights Imaging* **13**, 124 (2022).

17. Freeman, K. *et al.* Use of artificial intelligence for image analysis in breast cancer screening programmes: systematic review of test accuracy. *BMJ* **374**, n1872 (2021).
